# Viral and host mediators of non-suppressible HIV-1 viremia

**DOI:** 10.1038/s41591-023-02611-1

**Published:** 2023-11-13

**Authors:** Abbas Mohammadi, Behzad Etemad, Xin Zhang, Yijia Li, Gregory J. Bedwell, Radwa Sharaf, Autumn Kittilson, Meghan Melberg, Charles R. Crain, Anna K. Traunbauer, Colline Wong, Jesse Fajnzylber, Daniel P. Worrall, Alex Rosenthal, Hannah Jordan, Nikolaus Jilg, Clarety Kaseke, Francoise Giguel, Xiaodong Lian, Rinki Deo, Elisabeth Gillespie, Rida Chishti, Sara Abrha, Taylor Adams, Abigail Siagian, Dominic Dorazio, Peter L. Anderson, Steven G. Deeks, Michael M. Lederman, Sigal Yawetz, Daniel R. Kuritzkes, Mathias D. Lichterfeld, Scott Sieg, Athe Tsibris, Mary Carrington, Zabrina L. Brumme, Jose R. Castillo-Mancilla, Alan N. Engelman, Gaurav D. Gaiha, Jonathan Z. Li

**Affiliations:** 1 Brigham and Women’s Hospital, Harvard Medical School, Boston, MA USA; 2 Valley Health System, Las Vegas, NV USA; 3 Beijing Friendship Hospital Pinggu Campus, Capital Medical University, Beijing, China; 4 Massachusetts General Hospital, Harvard Medical School, Boston, MA USA; 5 University of Pittsburgh, Pittsburgh, PA USA; 6 Dana-Farber Cancer Institute, Harvard Medical School, Boston, MA USA; 7 Ragon Institute of MGH, MIT, and Harvard, Cambridge, MA USA; 8 Department of Biology, Massachusetts Institute of Technology, Cambridge, MA USA; 9 Division of Infectious Diseases and HIV Medicine, Department of Medicine, Case Western Reserve University/University Hospitals Cleveland Medical Center, Cleveland, OH USA; 10 Skaggs School of Pharmacy and Pharmaceutical Sciences, University of Colorado Anschutz Medical Campus, Aurora, CO USA; 11 Division of HIV, Infectious Diseases, and Global Medicine, University of California, San Francisco, CA USA; 12 Basic Science Program, Frederick National Laboratory for Cancer Research, National Cancer Institute, Frederick, MD USA; 13 Laboratory of Integrative Cancer Immunology, Center for Cancer Research, National Cancer Institute, Bethesda, MD USA; 14 Faculty of Health Sciences, Simon Fraser University, Burnaby, British Columbia Canada; 15 British Columbia Centre for Excellence in HIV/AIDS, Vancouver, British Columbia Canada; 16 Division of Infectious Diseases, Department of Medicine, University of Colorado Anschutz Medical Campus, Aurora, CO USA

**Keywords:** Translational research, HIV infections, Viral reservoirs, Virus-host interactions

## Abstract

Non-suppressible HIV-1 viremia (NSV) is defined as persistent low-level viremia on antiretroviral therapy (ART) without evidence of ART non-adherence or significant drug resistance. Unraveling the mechanisms behind NSV would broaden our understanding of HIV-1 persistence. Here we analyzed plasma virus sequences in eight ART-treated individuals with NSV (88% male) and show that they are composed of large clones without evidence of viral evolution over time in those with longitudinal samples. We defined proviruses that match plasma HIV-1 RNA sequences as ‘producer proviruses’, and those that did not as ‘non-producer proviruses’. Non-suppressible viremia arose from expanded clones of producer proviruses that were significantly larger than the genome-intact proviral reservoir of ART-suppressed individuals. Integration sites of producer proviruses were enriched in proximity to the activating H3K36me3 epigenetic mark. CD4^+^ T cells from participants with NSV demonstrated upregulation of anti-apoptotic genes and downregulation of pro-apoptotic and type I/II interferon-related pathways. Furthermore, participants with NSV showed significantly lower HIV-specific CD8^+^ T cell responses compared with untreated viremic controllers with similar viral loads. We identified potential critical host and viral mediators of NSV that may represent targets to disrupt HIV-1 persistence.

## Abstract (web summary)

Understanding the heterogeneity of HIV infection, such as in persons with non-suppressible HIV-1 viremia despite adherence to antiretroviral treatment, is crucial to better tailor therapeutic interventions to abrogate HIV-1 persistence.

## Main

For the majority of persons with human immunodeficiency virus (PWH), antiretroviral therapy (ART) suppresses HIV-1 RNA to below the level of commercial assay detection^1–5^. However, a subset of PWH demonstrate persistent low-level viremia while on ART^6,7^, a condition that not only represents a clinical conundrum for the clinician but can lead to substantial stigma and distress for the patient. Persistent low-level viremia has historically been attributed to suboptimal ART adherence and/or accumulating human immunodeficiency virus-1 (HIV-1) drug resistance^8,9^. Previous studies supporting the presence of active viral replication have reported that ART resistance mutations can accumulate when viremia persists in the low but detectable range^10,11^ and that low-level viremia can increase the risk of virologic failure^12^. However, there has been intriguing evidence that persistent low-level viremia can be maintained for long periods without leading to high-level virologic failure or the development of new resistance mutations^13–18^. This subset of individuals with persistent low-level viremia without evidence of ART non-adherence or meaningful drug resistance are defined as having non-suppressible HIV-1 viremia (NSV).

Clonal expansion of HIV-infected cells represents a key contributing factor for HIV-1 persistence, and recent studies have suggested that this plays an important role in NSV as well. Halvas et al. also studied a cohort of participants with NSV, reporting that the majority of plasma variants were composed of clusters of identical sequences without signs of viral evolution, which is consistent with the plasma viruses originating from a transcriptionally active viral reservoir rather than new rounds of infection^1^. While NSV was fueled by large populations of clonally expanded HIV-infected cells, the mechanisms that lead to the establishment and maintenance of NSV, and the NSV-generating proviral reservoirs, remain understudied. In this Article, we characterized a cohort of eight participants with NSV, and performed in-depth ART drug concentration testing, alongside viral and host cell genetics/genomics and immune profiling. We identified features of host integration sites that differentiated proviruses fueling NSV from those that were not contributory. Transcriptomic and immunologic phenotyping studies further highlight potentially permissive host cell and cellular immune environments in patients with NSV. These results provide the most comprehensive evaluation so far of the viral, cellular and immune mediators of NSV.

## Participant characteristics and assessment of ARV levels

We enrolled eight participants with ongoing NSV, 88% men, with a median age of 60 years and median ART duration of 10 years. The median duration of virologic suppression before the NSV and duration of NSV for all participants were 4 and 2 years, respectively. During the NSV episodes, the median viral load was 143 copies ml^−1^ and the median CD4 count was 798 cells µl^−1^ (Table 1 and Supplementary Table 1). Individual participant characteristics, ART regimens and genotypic susceptibility scores (GSS)^19^ of plasma viruses sequenced during NSV are presented in Supplementary Table 1. All participants were receiving at least two active antiretroviral drugs during the NSV episodes. Characteristics of the ART-suppressed and viremic controllers (VCs) historical comparator participants are presented in Supplementary Tables 2–4.

**Table 1:** Characteristics of NSV and ART-suppressed control participant cohorts

Characteristic		NSV (*n* = 8)	Control for HIV-1-specific CD8^+^ T cell (*n* = 7)	Control from ACTG (*n* = 11)
Sex, number (%)	Male	7 (88)	5 (71)	6 (42)
Age (years) (median [IQR^a^])		60 [55, 61]	62 [52, 68]	45 [40, 50]
Race/ethnicity (%)	African American	2 (25)	2 (29)	3 (27)
	Native American	1 (12)	0	0
	Caucasian	4 (50)	5 (71)	7 (63)
	Hispanic	1 (12)	0	1 (10)
Years on ART (median [IQR])		10 [7, 14]	12 [10, 13]	6 [5, 7]
Years with NSV^b^ (median [IQR])		4 [3, 9]	NA^d^	NA^d^
Years with suppression (median [IQR])		2 [1, 7]	12 [10, 13]	6 [5, 7]
Viral load copies ml^−1^ (median [IQR])		143 [87, 536]	BLD^e^	BLD^e^
CD4 count cells µl^−1^ (median [IQR])		798 [581, 1,008]	793 [617, 949]	806 [713, 1,208]

^a^IQR, interquartile range.

^b^NSV, non-suppressible HIV-1 residual viremia.

^c^Virologic suppression before the NSV.

^d^NA, not applicable.

^e^BLD, below level of detection (HIV-1 plasma viral below limit of detection).

We assessed ART adherence by quantifying antiretroviral (and their anabolites) drug concentrations in plasma or through dried blood spot (DBS) testing. LV1 and LV2 had plasma dolutegravir and darunavir concentrations consistent with ongoing ART use (Supplementary Table 5). LV3 and LV5-9 had DBS tests for tenofovir (TFV-DP, a measure of cumulative tenofovir/tenofovir alafenamide adherence)^20,21^ and emtricitabine (FTC-TP, a measure of recent emtricitabine dosing within the preceding 7 days)^22,23^. The median (range) FTC-TP level was 5 (4.4–6.7) pmol per punch and TFV-DP level was 3,702 (2,771–6,684) fmol per punch^24^. These concentrations are consistent with expected levels in ART-suppressed individuals, including the highest odds of suppression and lowest odds of future viremia^20,23,25,26^, suggesting that our participants with NSV had both high levels of short-term and cumulative ART adherence (Supplementary Fig. 1 and Supplementary Table 5).

## Plasma NSV were composed of large clones without evolution

HIV-1 integration targeting preferences are demarcated by various features of active chromatin, including transcription^27^, histone epigenetic marks^28^ and nuclear speckle proximity^29^. The reservoir landscape changes over time in response to ART and the host immune response to a quasi-homeostatic state marked by cell loss and clonal expansion^30–36^. A key goal of this study was to assess aspects of host proviruses that contributed to NSV. Longitudinal single-genome sequencing of near-full-length proviruses and plasma HIV-1 *pol*-*env* RNA was performed.

**Fig. 1:**
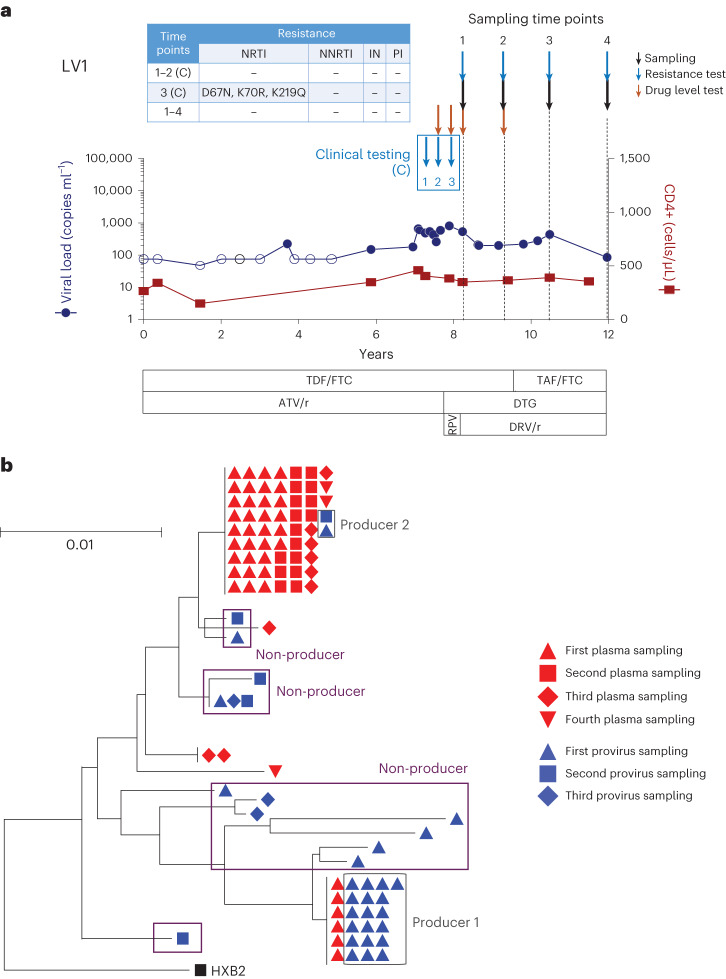
Example participant with non-suppressible viremia (LV1). **a**, Viral loads and CD4^+^ T cell count from the time of virologic suppression. Downward blue and orange arrows indicate timing of drug resistance and plasma drug level testing, respectively. Sampling times for viral genetic analyses are in black arrows. Antiretroviral resistance mutations from both clinical testing and the largest plasma clone from single-genome sequencing are shown in the table insert. The "(C)" denotes a clinical resistance testing result. **b**, Neighbor joining trees of proviral and plasma *pol-env* sequences in blue and red, respectively. Producers defined as proviruses with exact matches to plasma HIV-1 RNA sequences. Non-producers are proviruses that do not match any plasma HIV-1 RNA sequences. Shape indicates sampling time point, corresponding to black arrows in **a**. RPV, rilpivirine; TDF, tenofovir disoproxil fumarate; FTC, emtricitabine; ATV/r, atazanavir/ritonavir; TAF, tenofovir alafenamide; DTG, dolutegravir; DRV/r, darunavir/ritonavir; NRTI, nucleoside reverse transcriptase inhibitors; NNRTI, non-nucleoside reverse transcriptase inhibitors; IN, integrase; PI, protease inhibitor.

**Extended Data Fig. 1:**
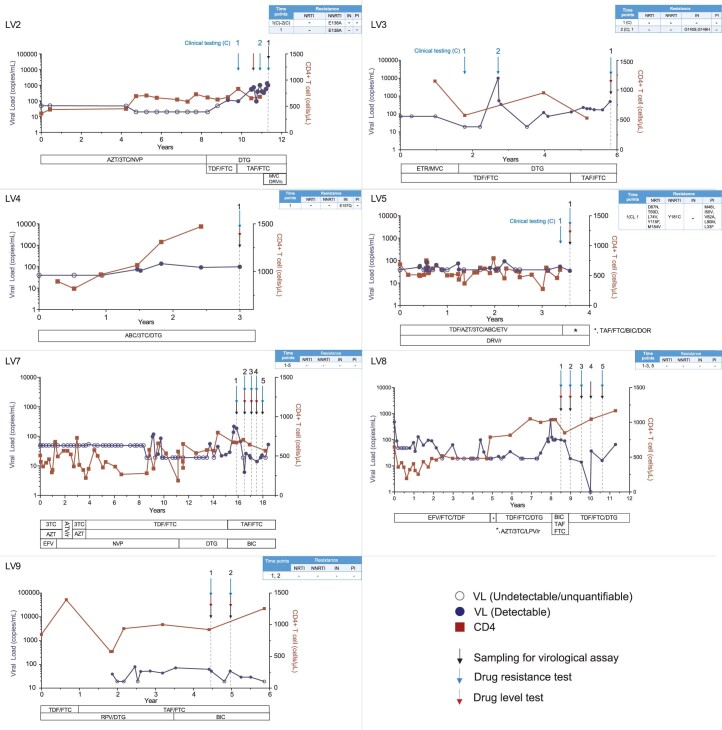
Viral load and CD4^+^ T cell count for seven NSV participants (Supplementary to Fig. 1). The table in each figure panel shows presence or absence of drug resistance mutations from both clinical testing and the largest plasma clone from single-genome sequencing. The "(C)" denotes a clinical resistance testing result.

A total of 1,987 single-genome proviral sequences and 222 single-genome plasma sequences were generated for the eight participants with NSV. Longitudinal plasma HIV-1 sequences were obtained for four participants with available sampling (LV1, LV7, LV8 and LV9), at a median 4.5 time points, an average of 9.7 months apart (Fig. 1a and Extended Data Fig. 1). While the ART regimen was adjusted during the NSV for some participants, virologic suppression was not achieved (Extended Data Fig. 1).

**Fig. 2:**
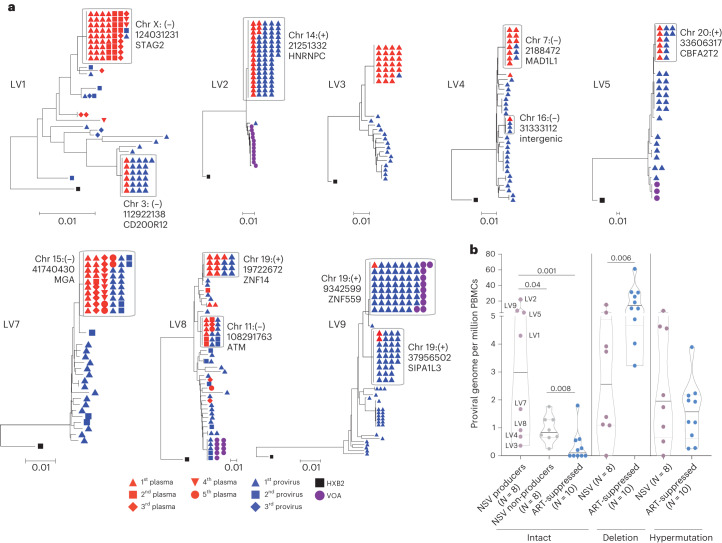
Sequencing overview of the non-suppressible viremia cohort. **a**, Neighbor joining trees show intact proviral and plasma sequences from different time points. The host integration sites of the producer proviruses are labeled. **b**, Comparison of reservoir size (number of proviral sequences per million cells) for producer proviruses versus non-producer proviruses in participants with NSV versus intact proviral reservoir size of ART-suppressed individuals. Median and interquartile ranges are labeled in the violin plot. Two-sided Wilcoxon matched-pairs signed-rank test and Mann–Whitney *U* tests were used for comparisons.

**Extended Data Fig. 2:**
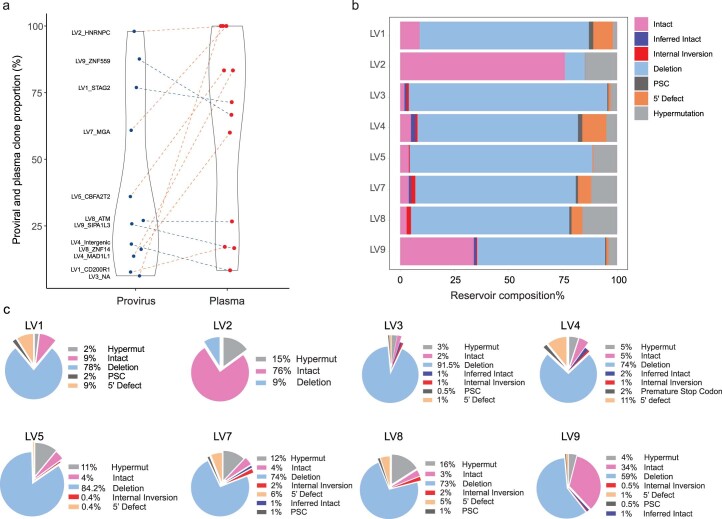
Reservoir size and composition for non-suppressible viremia participants (Supplementary to Fig. 2). (a) Comparison of proportion of the proviral and plasma HIV sequences comprised of each producer clone. Red dotted lines represent higher plasma clone frequency and blue dotted lines present higher proviral clone frequency. (b) and (c) Percentage of intact and defective proviral species for each NSV participant.

Phylogenetic analysis confirmed that sequences from each participant partitioned into separate clusters (Supplementary Fig. 2). We next looked for evidence of longitudinal HIV-1 sequence changes consistent with the active infection of new cells. Neighbor joining trees of proviral and plasma sequences for these eight participants instead showed that the plasma sequences were dominated by one or two clones, with no evidence of viral evolution, consistent with plasma virus originating from a transcriptionally active population of HIV-1-infected cells (Figs. 1b and 2a). For the initial analysis, proviral sequences were considered intact if they either did not harbor obvious defects or were linked to plasma sequences. At the time of study entry, the two largest plasma HIV-1 clones accounted for a median 71% (Q1–Q3: 27–83%) of all plasma sequences and were linked to a median 26% (Q1–Q3: 14–61%) of all intact proviral sequences (Extended Data Fig. 2a). Overall, intact proviruses accounted for a median 4.5% (Q1–Q3: 3.8–15%) of the proviral reservoir, with a high degree of variation evident from two participants (LV2 and LV9). LV2 and LV9 proviruses were dominated by several large clones of intact sequences that represented 76% and 34% of their total peripheral blood mononuclear cell (PBMC) proviral reservoirs, respectively (Extended Data Figs. 2b,c).

We categorized proviruses as producers if they matched a plasma sequence and as non-producers if they did not. There was a wide range of producer proviruses within the reservoir. For LV2, the PBMC proviral reservoir was largely composed of one large producer clone representing 98% of intact proviruses, which matched the large plasma NSV clone (Fig. 2a). In contrast, LV3 had the smallest producer reservoir size, representing 3.5% of total intact sequences. These results demonstrate that, while these individuals share a common NSV phenotype, their proviral landscape can be highly heterogenous (Extended Data Fig. 2b,c).

We next compared the size of the intact and defective reservoir sizes between the participants with NSV and a control group of ten ART-suppressed participants (Supplementary Table 2). Participants with NSV had a numerically larger total and significantly larger intact PBMC proviral reservoir (NSV versus ART-suppressed: median total proviral genomes 34 versus 18 proviruses per million cells, *P* = 0.08; and median intact proviral genomes 4.3 versus 0.1 proviruses per million cells, *P* = 0.001). Specifically, the size of the producer proviral reservoir was significantly larger in the participants with NSV than either the non-producer intact proviral reservoir in these participants or the intact proviral reservoir in the ART-suppressed participants (Fig. 2b). In addition, the participants with NSV had a smaller number of proviruses with large deletions (median 2.6 versus 10.7 proviruses per million cells, *P* = 0.006). These results suggest that an enlarged producer reservoir size could be a contributing factor to NSV.

## Integration site and epigenetic signatures of producers

**Fig. 3:**
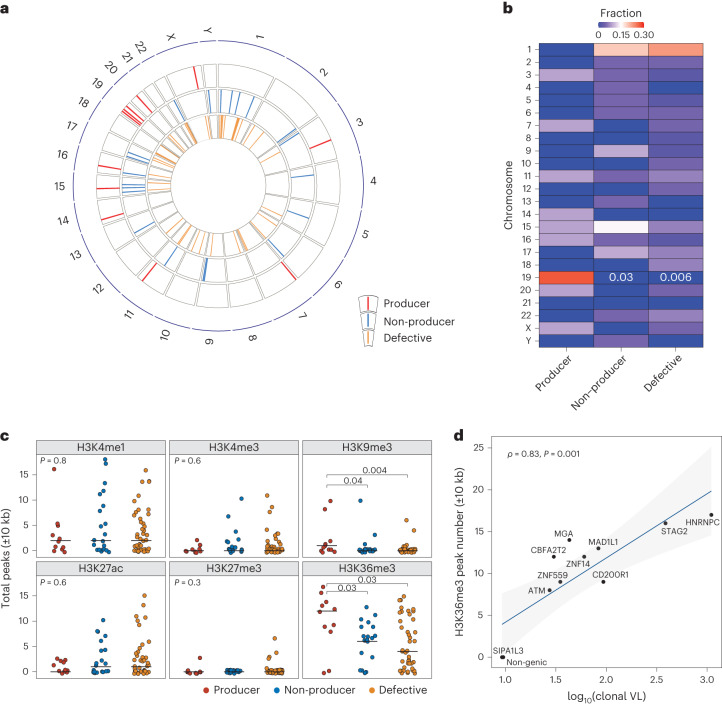
Integration sites and chromatin features of HIV-1 proviruses. **a**, Circos plot showing the location of each integration site across human chromosomes. **b**, Karyotyping heatmap showing the percentage of integration sites in each human chromosome for different classes of proviruses. Fisher’s exact test was used. **c**, Number of peaks for key histone marks in 10-kb regions flanking the proviral integration sites. Center lines represent medians. We used Tukey boxplots, in which boxes represent median values and first–third quartiles. Two-sided Kruskal–Wallis test was used to evaluate if there was significant differences among three groups and if so, two-sided Mann–Whitney *U* test was used to compare between-group differences. **d**, Correlation between enrichment of H3K36me3 histone marks near producer proviral integration sites and plasma clone viral loads (viral load multiplied by fraction of plasma sequences matching the producer provirus). Host gene integration sites are labeled. Two-sided Spearman correlation test was used. VL, viral load.

The location and chromatin landscape of HIV-1 proviral integration sites can modulate the extent of proviral transcriptional activity^37,38^. We accordingly evaluated whether certain integration site features differentiated the producer, non-producer and defective proviruses. Using the Matched Integration Site and Proviral Sequencing (MIP-seq) protocol^39^, we identified host chromosomal integration sites for 11 producer, 21 intact non-producer and 44 defective proviruses across all participants with NSV (we were unable to identify an integration site for the LV3 producer clone). Integration sites were identified across all autosomal and sex chromosomes with the exception of chromosome 21 (Fig. 3a). Compared with non-producer and defective proviruses, producer proviral integration sites were enriched in chromosome 19. Twenty-seven percent (3/11) of producer proviruses were located in chromosome 19 compared with none of the 21 non-producer and 44 defective proviruses (producer versus non-producer *P* = 0.03 and producer versus defective *P* = 0.006, Fig. 3b).

**Extended Data Fig. 3:**
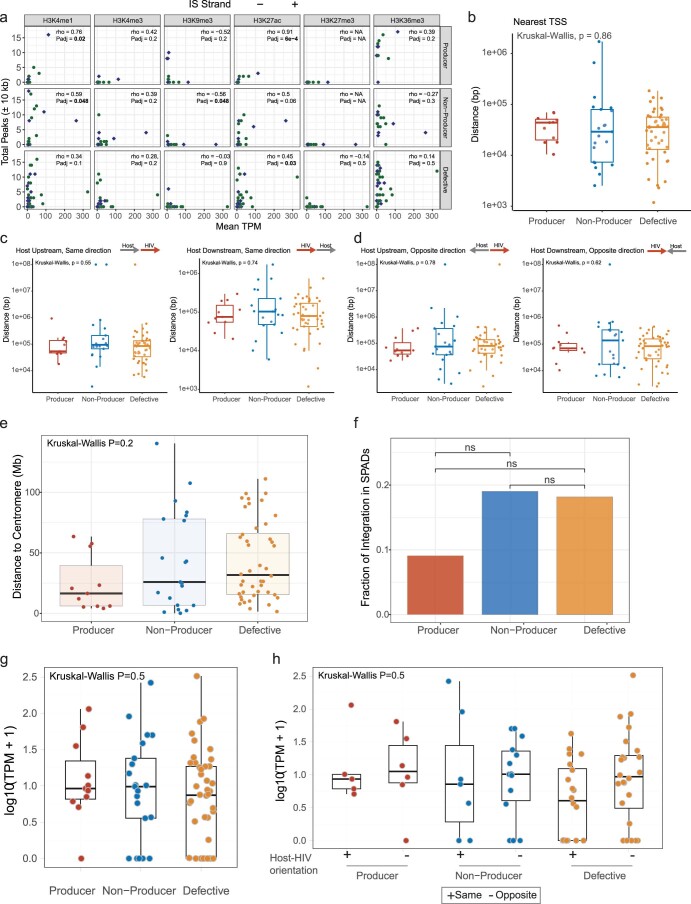
Integration site features (Supplementary to Fig. 3). (a) Correlation between enrichment of histone marks in producer integration sites and transcripts per million (TPM) from CD4^+^ T cell bulk RNA-seq results of each corresponding host gene harboring a provirus. On x-axis, total peak numbers in ± 10 kb flanking regions of n = 11 producer, n = 21 nonproducer and n = 44 defective integration sites from NSV samples were compared to each host gene TPM in their CD4 + T cells on y-axis. Two-sided Spearman correlation tests were used for the comparisons. (b) The distance of integration sites in each class of proviruses to the nearest transcriptional start sites (TSS). (c) The distance between the integration site to the TSS when HIV-1 or host genes are in the same orientation. (d) The distance between the integration site to the TSS when HIV-1 or host genes are in the opposite orientation. For figures b-d, n = 11 producer, n = 21 nonproducer and n = 44 defective integration sites from NSV samples were compared to each other. (e) The distance from the integration sites of different proviral classes to the centromere. Two-sided Kruskal-Wallis and Mann-Whitney U tests were used for comparisons. (f) Fraction of integration sites in speckle-associated domains (SPADs). Chi-squared test was used for comparisons. (g) Transcriptomic level of HIV host genes for producers, non-producers and defectives. For (e)-(g), n = 11 producer, n = 21 nonproducer and n = 44 defective integration sites from NSV samples were compared to each other. (h) Transcriptomic level of host genes for producers, non-producers and defectives categorized by directionality to closest host genes. We compared the number of transcripts per million cells for the host gene of each integrated provirus, including categorizing by the relative direction of the provirus to the host gene. “+” represents human and HIV genome in the same and “- “represents human and HIV in the opposite orientation of their transcriptions. Total of n = 11 producer (5 integrations site in the same and 6 in the opposite orientation), n = 21 nonproducer (7 integrations site in the same and 14 in the opposite orientation) and n = 44 defective (20 integrations site in the same and 24 in the opposite orientation) integration sites from NSV samples were compared to each other. All the boxplots in this figure are in the format of the Tukey boxplot, with the center line indicating median, box limits indicating upper and lower quartiles and the whiskers indicating 1.5x interquartile range. Two-sided Kruskal-Wallis or Mann-Whitney U tests was used for comparisons, if not otherwise specified. For P values, ns, not significant.

We also observed significant enrichment of producer proviruses in proximity to two epigenetic markers. Using chromatin immunoprecipitation followed by sequencing (ChIP–seq) data from primary CD4^+^ T cells published on the ROADMAP database^40^, we detected significantly higher ChIP–seq peak numbers for the H3K36me3 and H3K9me3 histone marks in proximity to producer integration sites compared with either non-producer or defective integration sites (Fig. 3c). We calculated the level of plasma viral load contributed by the producer provirus, which we designate as the plasma clone viral load. We observed a significant positive correlation between the number of H3K36me3 ChIP–seq reads in proximity to the producer proviruses integration sites and the plasma clone viral load (Spearman *r* = 0.83, *P* = 0.001, Fig. 3d). Proximity to H3K36me3 has been linked to proviral gene expression^38,41^, suggesting that producer proviruses are enriched near transcriptionally active regions of the chromosome and that producer proviruses could potentially leverage cellular transcriptional machinery for proviral expression and virion production^37^. In addition, a higher number of proximal ChIP–seq peak numbers for two other activating histone marks (H3K27ac and H3K4me1) were linked to greater expression of host genes containing integrated proviruses (Extended Data Fig. 3a), although these histone marks were not enriched near producer proviruses.

There were a number of chromosomal features that were not associated with the producer cell proviral phenotype. Distance to transcriptional start sites (TSSs) was statistically indistinguishable between producer, non-producer and defective proviruses, regardless of the orientation of the host gene and provirus (Extended Data Fig. 3b–d). We also did not detect any significant differences between producer, non-producer and defective proviral classes and their distance to heterochromatic centromeres or the fraction of integration into transcriptionally active speckle-associated domains (Extended Data Fig. 3e,f). Finally, using RNA-seq analysis from CD4^+^ T cells of participants with NSV, we detected no significant differences in host gene transcript levels between producer, non-producer and defective proviruses regardless of the integration orientation of the provirus with regard to the host gene (Extended Data Fig. 3g,h).

## Distinct cell survival and interferon signaling in NSV

**Fig. 4:**
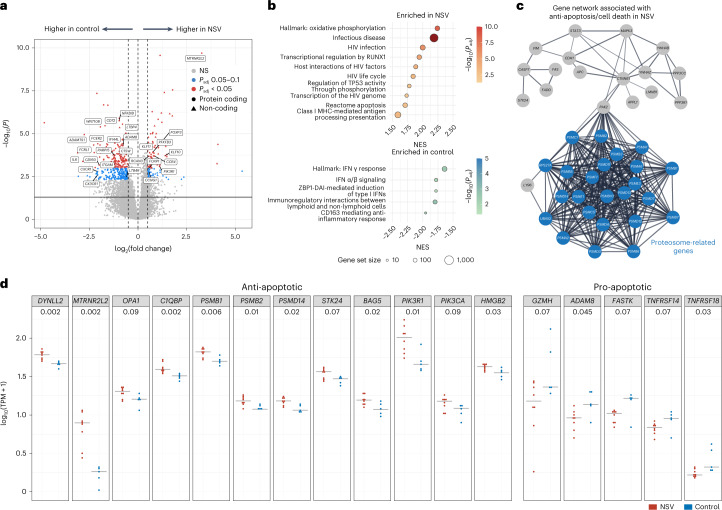
Transcriptomic analysis of CD4^+^ T cells from participants with NSV. **a**, Volcano plot shows DEGs in participants with NSV versus ART-suppressed individuals. Red and blue colors highlight differential statistical significance. Adjustments were made for multiple comparisons using the Benjamini–Hochberg method built in the DESeq2 package. **b**, Normalized enrichment score (NES) reflects the degree to which a set of genes is overrepresented among genes that are differentially expressed between participants with NSV and ART-suppressed control participants. Bar plot represents positively (red) and negatively (blue) correlated pathways. Adjustments were made for multiple comparisons using the Benjamini–Hochberg method. **c**, Genes related to apoptosis/cell death enriched in participants with NSV. **d**, Comparison of anti-apoptotic and pro-apoptotic gene transcription levels between NSV and ART-suppressed control group. Two-sided Mann–Whitney *U* tests were used for comparisons. TPM, transcripts per million.

**Extended Data Fig. 4:**
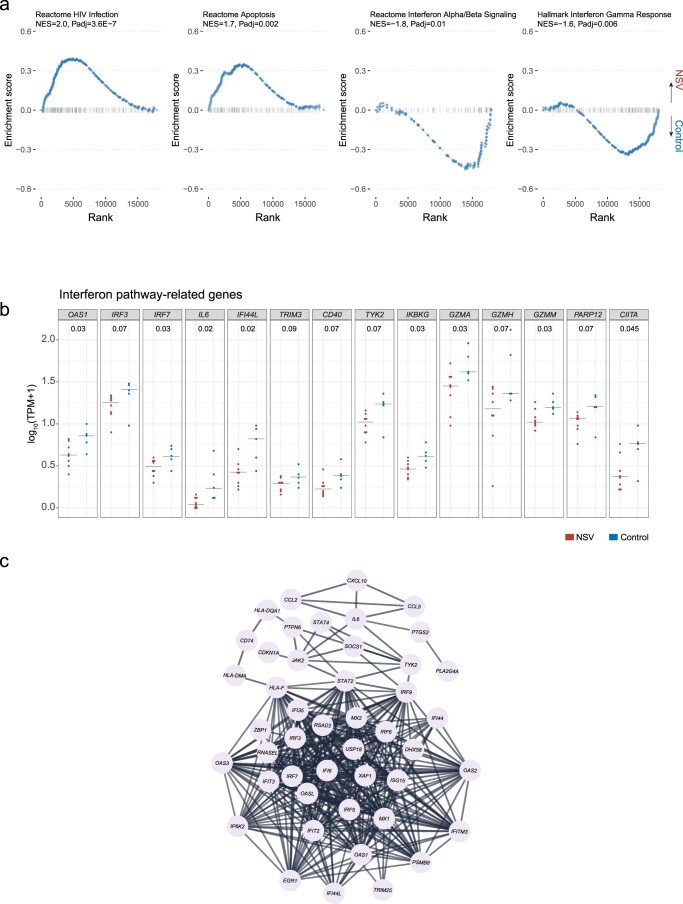
Transcriptomic analysis of CD4 + T cells from NSV and ART-suppressed participants (Supplementary to Fig. 4). (a) Gene Set Enrichment Analysis (GSEA) plots show selected pathways that were up- or down-regulated in NSV vs. ART-suppressed (control) groups. Adjustments were made for multiple comparisons using the Benjamini-Hochberg method. (b) Two-sided Wilcoxon rank sum test was used to evaluate interferon (IFN)-related gene expression levels between NSV and control groups. Bars represent median values. (c) Gene network related to IFN signaling enriched in the control group generated by STRING analysis.

Cell survival signaling has been linked to HIV-1 persistence, especially in latently infected CD4^+^ T cells^42,43^. To understand the association between cell signaling and NSV, we compared the CD4^+^ T cell transcriptomic features between the NSV group (*N* = 8) and a subgroup of the ART-suppressed individuals (*N* = 5) with available RNA sequencing (RNA-seq) data^44,45^. Compared with the ART-suppressed individuals, participants with NSV had 265 upregulated genes and 427 downregulated genes in total CD4^+^ T cells (adjusted *P* value (*P*_adj_) <0.1, Fig. 4a). Among these differentially expressed genes (DEGs), Gene Set Enrichment Analysis (GSEA) revealed enrichment of pathways related to HIV-1 infection, HIV-1 life cycle and transcription in the NSV group (Fig. 4b). CD4^+^ T cells from the NSV group exhibited enrichment in oxidative phosphorylation and apoptosis-related signals (Fig. 4b top and Extended Data Fig. 4a). Specifically, CD4^+^ T cells from participants with NSV appeared to be primed for survival via downregulation of pro-apoptotic genes and upregulation of genes associated with anti-apoptotic pathways, including proteosome-related genes (for example, *PSMB1*, *PSMB2* and *PSMD14*), ubiquitination-related genes, and oncogenes such as *PIK3CA* and *PIK3R1* (Fig. 4c,d)^46–49^.

Transcriptomic analysis also highlighted differences in the immune responses between NSV and ART-suppressed individuals. Participants with NSV demonstrated upregulation of immunosuppression-related genes in total CD4^+^ T cells, including *CTLA4* and *FOXP3* (Fig. 4a), pointing to an enrichment of the RUNX1-related pathway, which is associated with attenuation in antiviral and interferon (IFN) signaling through FOXP3 binding^50,51^. In fact, both IFN-α/β and IFN-γ signaling were enriched in ART-suppressed individuals (Fig. 4b bottom and Extended Data Fig. 4b,c). IFN signaling plays a pivotal role in HIV-1 pathogenesis by inducing viral restriction factors, causing depletion of CD4^+^ T cells, and regulating systemic immune activation^52^. These results may point towards potential defects in immune-mediated control of a highly active HIV-1 reservoir as a contributing factor for NSV.

## Absence of immune activation and evidence of HLA escape in NSV

**Fig. 5:**
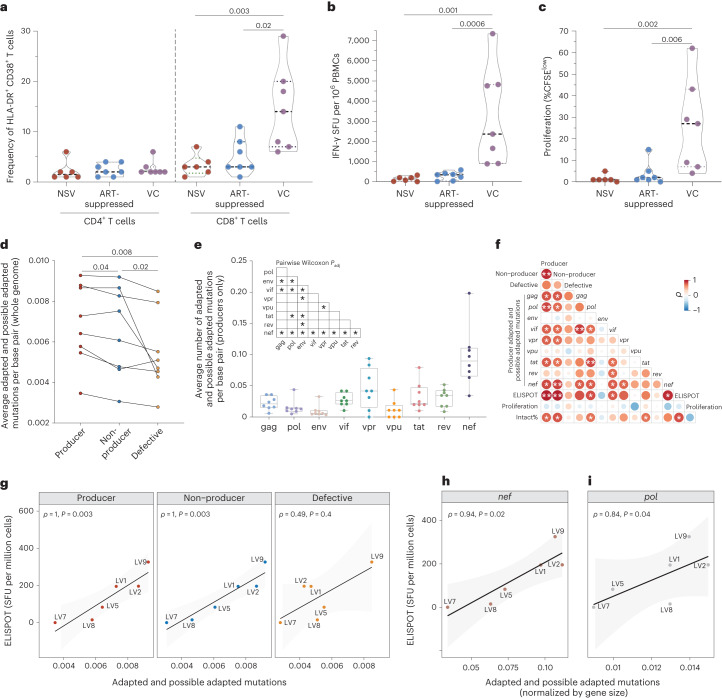
HIV-specific CD8^+^ T cell response and HLA class I escape mutations. **a**, Frequency of HLA-DR^+^CD38^+^ in CD4^+^ and CD8^+^ T cells. **b**, HIV-specific CD8^+^ T cell ELISPOT responses in NSV, ART-suppressed, and viremic controller (VC) cohorts. SFU, spot forming units. **c**, HIV-specific CD8^+^ T cell proliferation responses. Medians and interquartile ranges are shown in the violin plots. Two-sided Mann–Whitney *U* test was used. **d**, Average numbers of adapted and possible adapted HLA escape mutations per base across producer, non-producer and defective proviral sequences. Wilcoxon matched-pairs signed-rank testing was used. **e**, Average number of mutations per base pair for each HIV-1 gene in producer proviruses (*n* = 8). Two-sided pairwise Wilcoxon signed-rank test was used, and adjustments were made for multiple comparisons using the Benjamini–Hochberg method. In the boxplots, center lines indicated median, box limits indicated upper and lower quartiles and whiskers indicated minimal and maximal values. **f**, Spearman correlation between adapted and possible adapted mutations in different HIV-1 genes in producer proviruses alongside CD8^+^ T cell proliferation activity and percent intact provirus. **g**, Spearman correlation between adapted and possible adapted mutations in three proviral classes and CD8^+^ T cell activity (ELISPOT). **h**,**i**, Spearman correlation between CD8^+^ T cell activity (ELISPOT) versus average adapted and possible adapted mutations in *nef* (**h**) and *pol* (**i**) in producer proviruses (normalized for gene size). Spearman correlation test was used. NS, not significant; *P < 0.05, **P < 0.01.

**Extended Data Fig. 5:**
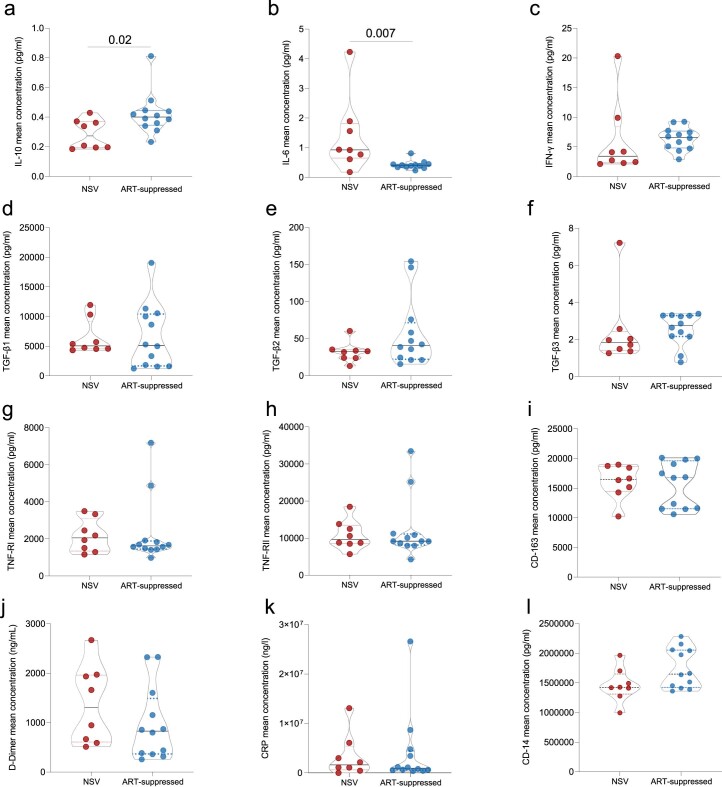
Soluble inflammatory markers in NSV versus ART-suppressed participants. (a) IL-10. (b) IL-6. (c) IFN-γ, (d) TGF-β1, (e) TGF-β2, (f) TGF-β3, (g) TNF-RI, (h) TNF-RII, (i) CD-163, (j) D-Dimer, (k) CRP and (l) soluble CD14 in NSV and ART-suppressed cohorts. Center lines indicated median levels and dotted lines indicated the first and third quartiles. The two-sided Mann-Whitney U test was used for comparisons.

**Extended Data Fig. 6:**
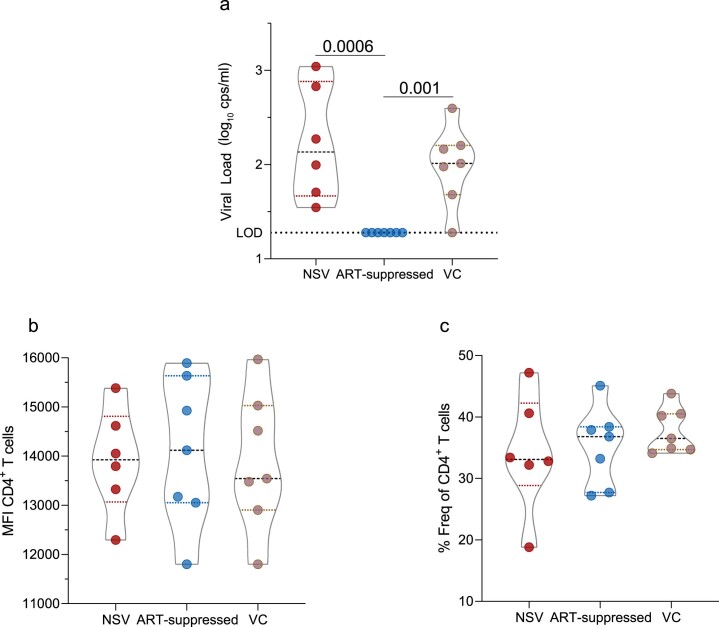
Viral load and CD4 + T cell features among NSV, ART-suppressed, and viremic controller cohorts. (a) Viral load of participants in NSV, ART-suppressed and viremic controller (VC) cohorts. (b) Mean fluorescence intensity (MFI) values of CD4 in NSV, ART-suppressed, and VC cohorts. (c) Frequency of CD4 + T cells. Center lines indicated median levels and dotted lines indicated the first and third quartiles. Two-sided Mann-Whitney U test was used.

We next assessed the impact of NSV on inflammation and immune activation. Levels of 12 plasma soluble markers of inflammation were compared between participants with NSV and ART-suppressed participants. Participants with NSV demonstrated lower IL-10 and elevated IL-6, but no significant differences in levels of C-reactive protein, sCD14 and sCD163 compared with ART-suppressed individuals (Extended Data Fig. 5). Of note, the older age of the participants with NSV could be playing a role in the higher IL-6 levels found in the participants with NSV compared with the ART-suppressed participants^53^. Levels of activated HLA-DR^+^CD38^+^ CD4^+^ and CD8^+^ T cells were compared among participants with NSV, ART-suppressed participants and a cohort of historical VCs, who can achieve immune-mediated control of HIV-1 replication without ART^54^. Despite similar levels of viremia to the VCs (Extended Data Fig. 6a), participants with NSV had substantially lower levels of CD8^+^ T cell activation compared with VCs and comparable levels compared with ART-suppressed individuals (Fig. 5a). There were no differences in the intensity and frequency of CD4-expressing cells between groups (Extended Data Fig. 6b,c).

**Extended Data Fig. 7:**
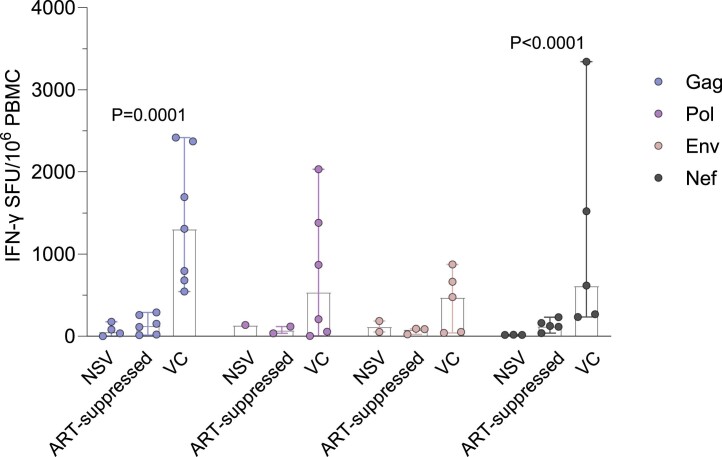
HIV-specific CD8^+^ T cell response among NSV, ART-suppressed, and viremic controller cohorts. For Gag-specific CD8^+^ T cell response, we had n = 4, n = 6 and n = 8 responses for NSV, ART-suppressed and VC samples, respectively. For Pol-specific response, we had n = 1, n = 2 and n = 7 responses from NSV, ART-suppressed and VC samples, respectively. For Env-specific response, we had n = 2, n = 3 and n = 6 responses from NSV, ART-suppressed and VC samples, respectively. For Nef-specific response, we had n = 3, n = 5 and n = 6 responses from NSV, ART-suppressed and VC samples, respectively. Bars represented median values and error bars indicated 95% confidence interval. Two-sided Kruskal-Wallis test was used to detect differences among three different cohorts. SFU, spot forming unit.

**Extended Data Fig. 8:**
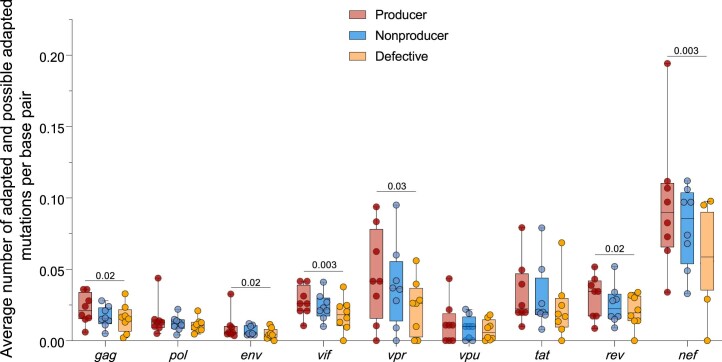
HLA class I escape mutations by different HIV-1 genes. Average number of adapted and possible adapted HLA escape mutations in producer, non-producer, and defective proviral sequences across the HIV-1 genome (normalized for gene size) among eight participants with NSV. Center lines in boxplots indicated median, box limits indicated upper and lower quartiles and the whiskers indicated minimal and maximal values. Friedman test was conducted within each gene area and Nemenyi post-hoc pair-wise tests were performed to evaluate the difference between two different proviral groups.

HIV-specific CD8^+^ T cells, which recognize viral peptides presented in complex with HLA Class-I (HLA-A, HLA-B, and HLA-C), are thought to be one of the key mediators of viral control^55,56^. Among NSV, ART-suppressed and VC participants, the magnitude of effector HLA-restricted HIV-specific T cell activity and proliferative HIV-specific CD8^+^ T cells aligned with overall CD8^+^ T cell activation. VCs had significantly greater HIV-specific CD8^+^ T cell responses by IFN-γ ELISpot and T cell proliferation assays in comparison with both participants with NSV and ART-suppressed participants (Fig. 5b,c). In VCs, elevated HIV-specific CD8^+^ T cell responses were detected against HIV-1 Gag, Pol, Env and Nef peptides, with the most robust responses towards Gag (Extended Data Fig. 7). Of note, the relatively muted HIV-specific CD8^+^ T cell response in NSV was paired with a modestly higher average number of HLA-adapted (escape) mutations^57^ in producer proviruses compared with non-producer (*P* = 0.04) and a substantially higher escape burden compared with defective proviruses adjusted for proviral length (*P* = 0.001, Fig. 5d). After normalizing for the size of each HIV-1 gene, *nef* showed significantly higher numbers of adapted and possible adapted mutations compared with other HIV-1 genes (Fig. 5e and Extended Data Fig. 8). Adapted and possible adapted mutations in HIV-1 genomes in both producers and non-producers highly correlated with CD8^+^ T cell IFN-γ release but not with proliferation (Fig. 5f,g), suggesting a relationship between effector HIV-specific CD8^+^ T cell responses with relatively decreased functionality (that is, proliferation) and subsequent emergence of mutations within proviral clones. Specifically, the number of *nef* adapted and possible adapted mutations in producer proviruses was strongly correlated with CD8^+^ T cell IFN-γ release in NSV (*r* = 0.94, *P* = 0.02, Fig. 5f,h). Adapted and possible adapted mutations in *pol* in producer proviruses also significantly correlated with total CD8^+^ T cell activity (*r* = 0.84, *P* = 0.04, Fig. 5i) and may represent immune-driven viral escape mutations that accumulated before ART initiation.

## A subset of producers has deletions in the 5′ leader region

**Extended Data Fig. 9:**
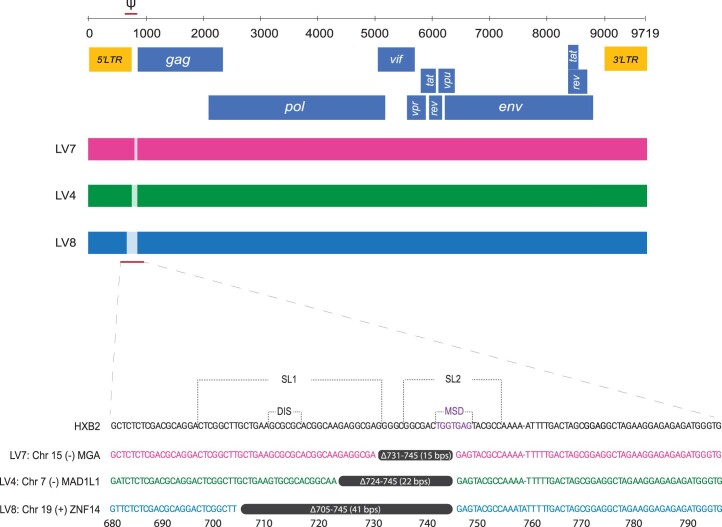
Location of deletions in the 5’ leader sequence in three NSV participants. Deletions are shown in the black regions at the bottom. Ψ, PSI element; SL1, stem loop; SL2, stem loop; DIS, dimerization initiation site; MSD, major splicing donor.

**Extended Data Fig. 10:**
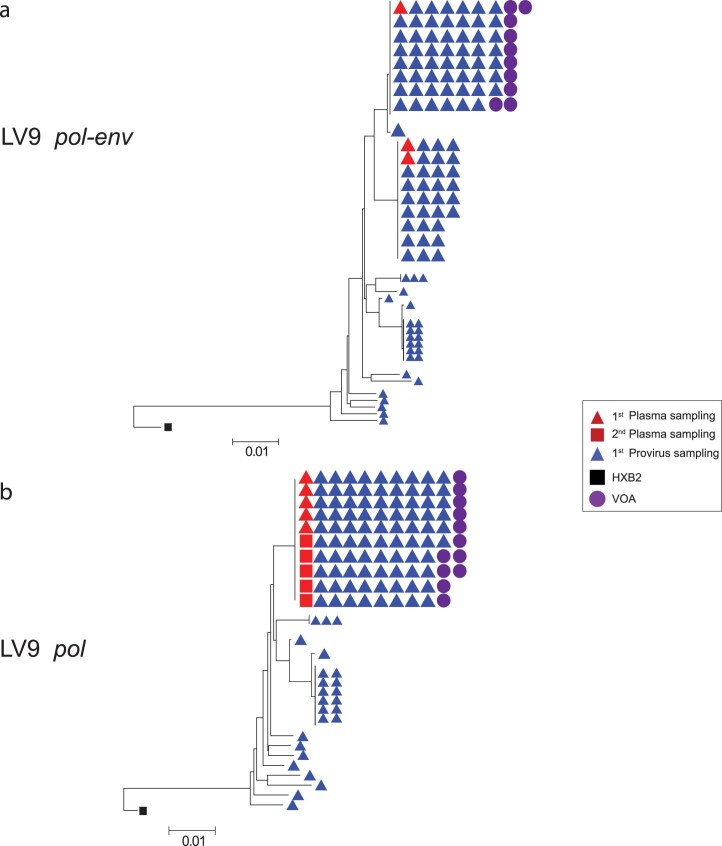
Neighbor joining trees of LV9 trimmed either to the pol-env or pol regions. (a) Proviral and plasma sequences trimmed to the pol-env region. This panel is reproduced from Fig. 2a. (b) Proviral and plasma sequences trimmed on the pol region alone showing an inability to discriminate between two clonal populations seen with pol-env sequencing.

Our sequencing revealed that NSV is largely composed of one or two clonal populations that remain stable over time, which is consistent with high-level viral production from a large, clonally expanded population of HIV-1-infected cells as the primary driver of NSV, rather than ongoing viral replication on ART. Since NSV need not be composed of infectious virus, we evaluated producer proviruses for potential replication defects, including deletions in the 5′ PSI packaging element^58^. In 38% (3/8) of participants with NSV, we observed that producer proviruses harbored deletions in the 5′ end of HIV-1 genome (Extended Data Fig. 9, black boxes). These deletions, which encompassed 22, 15 and 41 nucleotides in participants LV4, LV7 and LV8, respectively, all occurred within SL1 and SL2 elements, ending at the same location within the splice donor site. Plasma HIV-1 RNA sequencing of the 5′ leader/*gag* region of HIV-1 was performed to confirm the presence of these 5′ defects within the plasma HIV-1 RNA sequences. To evaluate whether these proviruses were infectious, viral outgrowth assays (VOAs) were performed using a transwell system with participant CD4^+^ T cells (LV4, LV7 and LV8; LV2, LV5 and LV9 served as controls) in the bottom chamber and MOLT-4/CCR5 cells in the upper chamber. HIV-1 DNA from the MOLT-4 cells was extracted and subjected to MIP-seq analysis. None of the proviruses with 5′ deletions was isolated in the VOA. Producer provirus was isolated from the VOA for LV9 (Fig. 2a and Extended Data Fig. 10). Non-producer proviruses were isolated from the VOA for LV2, LV5 and LV8 (Fig. 2a)^58^.

## Discussion

In this study, we have conducted a comprehensive assessment of NSV in eight participants and have provided insight into ART-independent factors implicated in HIV-1 suppression and persistence. Our results indicate that suboptimal ART adherence and drug resistance do not appear to be the drivers of non-suppressible viremia. In these participants, our data suggest that NSV is driven instead by the critical intersection of viral and host immune factors. Specifically, the NSV phenotype was highlighted by the presence of large, clonally expanded reservoirs of proviruses frequently harboring immune escape mutations (and/or defects in the 5′ leader region), integrated in transcriptionally permissive chromosomal regions, within CD4^+^ T cells primed for survival, and in an environment of muted HIV-specific T cell responses (Supplementary Fig. 3).

In one of the first in-depth reservoir studies of NSVs, Halvas et al. reported that NSV is composed largely of identical populations of plasma viruses that arise from the expansion of HIV-infected CD4^+^ T cell clones, which they termed repliclones^1^. However, most prior studies sequenced relatively short fragments of viral RNA, which can overestimate the clonality of plasma sequences. Our plasma HIV-1 RNA sequencing assay was combined with an ultrasensitive RNA extraction process for 6.7 kb *pol-env* RNA-seq (Extended Data Fig. 10). These results confirm that, in our cohort, NSV is composed primarily of one to two large plasma viral clones that composed >70% of plasma viruses. The role of these viral clones as the primary driver of NSV was confirmed across multiple longitudinal time points, which failed to reveal evidence of plasma viral sequence changes and evolution. For all of the participants with NSV, we were able to identify exact proviral sequence matches for the large plasma clones. We found that the size of the producer proviral reservoir was significantly larger than either the size of the non-producer proviruses in participants with NSV or intact proviruses in ART-suppressed participants, although admittedly with a broad distribution in size of the producer proviral reservoir. The large size of these producer proviral reservoirs and their ability to maintain NSV over years highlight the relative stability of this reservoir. These results and the presence of NSV over many years suggests an intrinsic ability of these HIV-infected cells to maintain prolonged survival and/or proliferate. Prior studies have reported that CD4^+^ T cells modulating key pro- and anti-apoptotic pathways can maintain survival of HIV-infected cells, drive clonal expansion and guard against CTLs^43,59^. Compared with ART-suppressed participants, CD4^+^ T cells in participants with NSV demonstrated transcriptional upregulation of anti-apoptotic pathways and downregulation of pro-apoptotic pathways. While transcriptional analysis was not isolated to producer cells alone, these results suggest that the CD4^+^ T cell environment in participants with NSV is primed for survival. Lymphoid clonal hematopoiesis (L-CH) refers to the expansion of hematopoietic stem cell clones in relation to age that is triggered by the acquisition of specific point mutations or chromosomal alterations in somatic cells. Recent studies have revealed the presence of L-CH in conditions such as autoimmunity or immunodeficiency, and the role of L-CH in NSV should be studied in the future^60^.

Using the MIP-seq assay^39^, we were able to identify the location of HIV-1 integration sites in host chromosomes for 11 producer, 21 intact non-producer and 44 defective proviruses across participants with NSV. HIV-1 integration is known to favor active chromatin, and proximity to activating epigenetic marks can modulate proviral gene expression and hence the fate of the resident provirus and infected cell^61,62^. We and others have reported that with prolonged ART, intact proviruses are more likely to be found in non-genic regions, in opposite orientation from the host gene and more distant from accessible chromosomal regions that highlight the selection of intact proviruses integrated into regions of deep latency^63^. In participants with NSV, we found enrichment of integration in chromosome 19, which is distinctively enriched for gene density^64,65^. We also demonstrated that producer proviruses were located in regions enriched in certain epigenetic characteristics, including a greater number of H3K36me3 histone peaks, which are associated with a transcriptionally permissive chromosomal regions and elevated proviral expression^38^. A higher number of H3K36me3 peaks surrounding the producer provirus was also strongly associated with higher plasma viral load composed of that clone. These results show that the location of HIV-1 proviral integration in the host genome is a determinant of its eventual fate and align with emerging evidence that the processes of entering and exiting proviral transcriptional latency are influenced by epigenetic mechanisms, making them potentially susceptible to pharmacological interventions.

We found that the persistence of non-suppressible viremia occurred in an environment of relatively muted inflammation and immune activation, featuring the downregulation of IFN response genes, generally low levels of soluble inflammatory markers, and no significant increase in HIV-specific CD8^+^ T cell responses compared with ART-suppressed individuals. IFN plays a vital role in the innate host antiviral response and contributes to the suppression of HIV-1 viremia^66^. Compared with ART-suppressed participants, participants with NSV significantly downregulated transcription of multiple genes involved in the IFN-response pathway by bulk CD4 transcriptomic analysis. These include IFN regulatory factors 3 and 7 (*IRF3* and *IRF7*), *OAS1* and other genes known to be part of critical circuits for stimulating host antiviral immune responses^67,68^. While transcriptomic analysis was performed only on bulk CD4 cells, the results do provide some intriguing signals related to latency reversal and cellular survival. For example, BIRC2 is an inhibitor of apoptosis protein (IAP), which suppresses HIV-1 transcription and depletion of BIRC2 can lead to HIV-1 latency reversal^69^. Participants with NSV downregulated the expression of TNFRSF14, the receptor for the LIGHT/TNFSF14 ligand, which binds BIRC2 (ref. ^70^). Inhibitors of LIGHT/TNFSF14 are being developed for cancer immunotherapy^71^ and could be assessed for HIV-1 latency reversal potential. In addition, participants with NSV were found to upregulate expression of PIK3R1 and PIK3CA (among other anti-apoptotic genes), which help encode phosphatidylinositol 3-kinase, an enzyme that promotes cellular survival through activation of Akt. Whether the use of phosphatidylinositol 3-kinase inhibitors could contribute to clearing HIV-infected cells should also be explored^72,73^.

Heightened HIV-specific CD8^+^ T cell responses occur with HIV-1 viremia, which is critical to suppress the HIV-1 reservoir^74^. Thus, we were surprised to find a relatively muted CD8^+^ T cell response, with no significant differences noted in HLA-restricted HIV-specific CD8^+^ T cell activity and proliferation between the individuals with NSV and ART-suppressed individuals, and significantly lower levels than seen in VCs with similar levels of viremia. Together, the transcriptomic, soluble inflammatory and T cell data demonstrated that NSV appears to be uncoupled with immune activation. There are several possible explanations for the relatively low levels of HIV-specific T cell responses. First, producer proviruses harbored higher frequencies of adaptive HLA escape mutations, which may explain the suboptimal induction of HIV-specific CD8^+^ T cell activity due to a loss of antigen recognition^75^. Of note, there was an enrichment of HLA-escape mutations in *nef*, which may be particularly immunogenic as previous studies have reported that the strength of the Nef-specific T cell activity is linked with the size of the HIV-1 reservoir^76^. Another potential mechanism of immune escape may be the contribution of defective virions to NSV. In 38% (three of eight) of our individuals with NSV, we detected deletions in the 5′ leader sequence of the HIV-1 genome. None of these sequences was recovered using the VOA, suggesting these proviruses may be replication defective. The 5′-untranslated leader contains several structural motifs that are involved in multiple steps of HIV-1 replication. The deletions are present in the psi (Ψ) element, which is a highly structured RNA sequence with four hairpin stem loops and a strong affinity for the nucleocapsid domain of the viral Gag protein. Genome packaging during virus assembly and reverse transcription during the subsequent round of infection are some known functions of the 5′ leader region^77,78^. A recent study by White et al. described four participants with NSV with apparent defects in the 5′ leader sequence^58^. These defects generally spanned the major splice donor site and resulted in the creation of nonfunctional virions lacking the envelope glycoprotein. Interestingly, the 5′ leader sequence deletions in our participants with NSV spanned the same region and, in fact, LV4 shares the same 22-base deletion that was detected in three participants by White et al. The detection of 5′ leader defects at high frequencies across multiple cohorts suggests a selective advantage of these proviruses in conferring the NSV phenotype, potentially by maintaining a plasma viral load in the absence of HIV-1 replication and/or the ability of Env-deleted virions to escape from host immune surveillance^79^. It has also become increasingly clear that cellular metabolism represents a crucial regulator of T cell activation and response. Additional studies are also needed to assess the role of metabolic programs and pathways, such as altered glutaminolysis and glycolysis, in suppressing T cell function in participants with NSV^80^.

In addition to the previously noted limitations, the small size of the NSV cohort analyzed in this study is a key limitation. Also, we estimated proviral reservoir size by near-full-length proviral sequencing. This could lead to some underestimation of the actual reservoir size, although other methods for reservoir quantification (for example, intact proviral DNA assay) may overestimate the size^81^. Future studies will need to investigate the size of producer proviruses within tissue reservoirs. We found that the peripheral blood reservoir of HIV-infected CD4^+^ T cells contributing to NSV can differ dramatically between participants with NSV. It is possible that NSV-generating CD4^+^ T cells are also distributed within anatomical tissue compartments^82,83^, especially for those participants with NSV with a relatively small producer proviral reservoir size in the peripheral blood. Prior studies have shown that Gag- or CMV-specific antigens can drive the expansion of certain HIV-infected cellular clones^32^. Additional studies are needed to delineate which antigens might be playing a role in the expansion of the producer proviruses. Neutralizing antibody responses can also suppress viremia^84^ and evaluation of the humoral immune responses are indicated, although prior studies suggest that some NSV is resistant to autologous neutralizing antibodies^85^. The lack of viral evolution and ongoing viral replication on longitudinal phylogenetic analysis may explain why ART intensification has not led to virologic efficacy in those with NSV. It is unknown whether newer ARVs, including drugs with potential immunomodulatory properties (for example, fostemsavir^86^) could provide additional virologic benefit.

In this study, we identified critical host and viral mediators of NSV that represent potential targets to disrupt HIV-1 persistence and promote viral silencing. Importantly, ultrasensitive HIV-1 viral load assays can detect residual low levels of HIV-1 viremia in the vast majority of PWH, even on apparently suppressive ART^87^. Previous studies have reported that such residual viremia is largely composed of drug-sensitive virus^16^ and relatively homogeneous viral populations^18^. Thus, we believe it is likely that the mechanisms behind NSV that we describe here are present to some extent in most, if not all, of PWH. Achieving an in-depth understanding of the mechanisms behind NSV may provide insight on strategies for HIV-1 reservoir eradication applicable to all PWH.

## Methods

### Participants

In an observational study, we enrolled 8 ART-treated participants with three or more HIV-1 RNA levels between 40 and 1,000 copies ml^−1^ over 24 months of whom 7 (88%) were men and compared them with a historic cohort of 11 ART-suppressed participants, of whom 5 (71%) were men, with HIV-1 with similar demographic and CD4 counts. Sex and/or gender of participants was determined on the basis of self-report. A participant with non-suppressible viremia enrolled in the HIV Eradication and Latency (HEAL) cohort, a biorepository of Brigham and Women’s Hospital, was included as a part of eight NSVs. The NSV samples were taken from different time points, enabling us to study these participants longitudinally (Fig. 1 and Extended Data Fig. 1). The ART-suppressed comparators included 11 participants from the AIDS Clinical Trials Group (ACTG) and 7 participants from the Ragon Institute of Massachusetts General Hospital, the Massachusetts Institute of Technology and Harvard. Both ART-suppressed comparator groups were well matched with the participants with NSV by CD4 count. A cohort of VCs not on ART with similar viral loads to the participants with NSV was also included for the T cell analysis. All study participants provided written informed consent. The study was approved by the Mass General Brigham Institutional Review Board.

### ARV drug level testing

For plasma ARV testing, samples were sent to the infectious disease pharmacokinetics lab at the University of Florida. Testing was performed for darunavir and dolutegravir by liquid chromatography with tandem mass spectrometry. For DBS ARV testing, 25 ml of whole blood were spotted five times onto Whatman 903 protein saver cards, as previously described^21,82^. After spotting, cards were allowed to dry at room temperature for at least 3 h (as long as overnight), after which they were stored at −80 °C until analyzed. TFV-DP and FTC-TP were quantified from two 7-mm punches extracted with 2 ml of methanol:water to create a lysed cellular matrix using a previously validated method that was adapted and validated for tenofovir alafenamide-containing regimens. The assay was linear and ranged from 25 to 6,000 fmol per sample for TFV-DP and from 0.1 to 200 pmol per sample for FTC-TP^21,88^.

### DNA isolation and HIV reservoir quantification

DNA extractions were carried out from PBMCs using the QIAamp DNA Micro Kit (cat. no. 56304), and the quantification of DNA was performed with Nanodrop (Applied Biosystems, Thermo Fisher). To estimate the size of the reservoir, we analyzed near-full-length proviral sequences as described below.

### Near-full-length proviral sequencing, sequence alignments, quality control and neighbor joining analyses

Extracted DNA was endpoint-diluted and subjected to near full-length sequencing (NFL-seq), as previously described^63^. We classified our sequences into intact and different classes of defective proviruses (for example, 5′-defect, deletion, hypermutation and inversion) using a published proviral intactness pipeline^88^. Briefly, after aligning to HXB2, we called our sequences as large deleterious deletions if they had a <8,000-bp amplicon size, out-of-frame indels, premature/lethal stop codons, internal inversions or packaging signal deletions (≥15 bp). The Los Alamos National Laboratory HIV Sequence Database Hypermut 2 program was used to identify the existence or nonexistence of hypermutations linked to APOBEC-3G/3F. Sequences of the virus that did not have any of the mutations listed earlier were categorized as ‘genome-intact’ sequences. Using MAFFT v7.2.0, we aligned the sequences and utilized MEGA 6 to deduce neighbor joining trees. We called those proviruses with an exact match with plasma sequences ‘producers’ and intact proviruses without matching plasma sequences ‘non-producers’.

### Plasma HIV-1 RNA sequencing

We sequenced plasma HIV RNA as previously described.^89^ Extracted RNA was diluted to single viral genome levels to meet the criteria of single-genome sequencing of having no more than one template in each well, theoretically no more than 25% of wells being positive for HIV. Primers were designed to amplify *pol-env*, a 6.7-kb region (Supplementary Data 1). The amplification reaction was performed using 0.5 µl primers (10 µmol), 1 µl (10 mmol) MgSO_4_, 1 µl (10 mmol) dNTPs and 1 U Platinum Taq Polymerase (Invitrogen) in 25 µl total volume. Polymerase chain reaction conditions consisted of a denaturation step at 94 °C for 2 min, followed by 30 cycles of 30 s at 94 °C, 30 s at 56 °C, 90 s at 68 °C and 10 min at 68 °C. Products underwent Illumina barcoded library construction and MiSeq sequencing. Amplicons were assembled using the UltraCycler v1.0 automated de novo sequence assembly to generate a continuous fragment. Plasma sequences that were within one to two nucleotides of the near-full-length proviral sequence was considered part of the clonal cluster. We counted the total number of plasma sequences in each clone and divided them by all plasma sequences that we had generated. Then we multiplied the ratio with the plasma viral load to determine the contribution of each clone for plasma viral load, which we termed the plasma clone viral load.

For each sequence, the GSS versus the participants’ ART regimen was calculated using the Stanford HIV database drug resistance scoring system. The Stanford HIV database provides a weighted penalty score for the effect of every resistance mutation and antiretroviral medication with 0 if there is no expected effect to 60 for high-level resistance. For each sequence, the estimated level of resistance for each antiretroviral medication (ARV) was determined by adding all of the penalty scores for each of the drug resistance mutations present. The GSS of each ARV was defined as the following: 1 (Stanford penalty score 0–9), 0.75 (Stanford penalty score 10–14), 0.5 (Stanford penalty score 15–29), 0.25 (Stanford penalty score 30–59) and 0 (Stanford penalty score ≥60). The GSS for the sequence was the sum of the GSS for each ARV as part of the participant’s regimen.

### Total RNA transcripts sequencing

CD4^+^ T cells were selected from cryopreserved PBMCs using EasySep Human CD4^+^ T Cell Enrichment Kit (STEMCELL Technologies). RNA was extracted from selected CD4^+^ T cells with the AllPrep DNA/RNA kit (Qiagen) with subsequent ribosomal RNA depletion RNA reverse transcribed to complementary DNA library and sequenced by NovaSeq (Illumina). Sequencing results were processed with the VIPER pipeline for alignment, counting and quality control^90^. DEG analysis was performed with DESeq2 package^89^ and GSEA with fgsea package using the adaptive multilevel splitting Monte Carlo approach (*n* = 10,000 for simple fgsea in preliminary estimation of *P* values)^91^.

### Integration site identification and epigenetics

We characterized single proviral genomes along with their matched genomic integration sites by MIP-seq^39^. Briefly, we initiated whole-genome amplification by performing multiple displacement amplification with phi29 polymerase using the QIAGEN REPLI-g Single Cell Kit, following the manufacturer’s protocol. Afterward, we divided DNA from each sample and carried out proviral sequencing and integration site analysis. We utilized integration site loop amplification, which has been previously described, to obtain the integration sites associated with each viral sequence^92^. One modification that we made to this assay is targeting both the 5′ and 3′ ends of HIV to assess the integration site on both ends and eliminate any potential bias that may arise from analyzing only one end of HIV. To determine the exact location of HIV in the host gene, we used an online tool for trimming integration sites^93,94^. We analyzed our integration sites for various histone marks by utilizing ChIP–seq datasets from primary CD4^+^ T cells that were publicly available on the ROADMAP website^40^. The NIH Roadmap Epigenomics Mapping Consortium produces a public resource of human epigenomic data to catalyze basic biology and disease-oriented research (http://www.roadmapepigenomics.org/). We calculated the total number of peaks of histone marks in a 10-kb window from the flanking sides of the integration site and regarded it as the total peak number. To determine the distance between the IS and the nearest TSS, we employed ‘nearestTSS: Find Nearest Transcriptional Start Site’, which is a tool developed in included in the edgeR package^95^.

### Inflammatory soluble marker levels

Cryopreserved plasma samples were thawed and analyzed in batch to assess inflammatory cytokines. Assays were run on 96-well plates. Duplicates were performed for 25% of the samples that were run on each plate. Mesoscale Discovery single and multiplex assays were performed according to instructions provided in kits for assessment of sCD14 (F21T1-2), sCD163 (F21J4-3), TNF-RI (R210V-3), TNF-RII (F21ZS-3), IFN-γ, IL-6, IL-10, IP-10, C-reactive protein (K151A9H-1) and TGF-β1-3 (K15241K-1). D-dimer levels were measured with a standard enzyme-linked immunosorbent assay (Diagnostica Stago S.A.S.). The Mesoscale assays were analyzed with a MESO QuickPlex SQ 120MM Reader.

### T cell activation phenotyping and assessment of surface CD4^+^ expression

PBMCs were thawed and stained with Live/Dead Violet (Thermo Fisher, 1:1,000), PE-Cy7-CD3 (clone SK7, BioLegend, 1:100), BV711-anti-CD4 (clone RPA-T4, BioLegend, 1:100), APC-anti-CD8 (clone SK1, BioLegend, 1:100), Alexa Fluor 700-anti-CD25 (clone M-A251, BioLegend, 1:50), BV650-anti-CD38 (clone HB-7, BioLegend, 1:50), FITC-anti-CD69 (FN50, BioLegend, 1:50), BV785-anti-HLA-DR (clone L243, BioLegend, 1:50) and PE/Dazzle 594-anti-PD-1 (clone EH12.2H7, BioLegend, 1:100). Cells were washed and fixed in 2% paraformaldehyde before flow cytometric analysis on a BD LSR Fortessa (BD Biosciences). CD4 surface expression was determined by assessment of CD4 mean fluorescence intensity.

### Assessment of HIV-specific CD8^+^ T cell reactivity

PBMCs were resuspended at 1 × 10^6^ ml^−1^ in RPMI supplemented with 10% fetal bovine serum (R10) and plated 200 µl per well in Immobilon-P 96-well microtiter plates (Millipore) precoated with 2 µg ml^−1^ anti-IFN-γ (clone DK1, Mabtech). Individual HLA-restricted HIV peptides from the optimal A-list^96^ matched to each subject’s HLA genotype were added at 1 µg ml^−1^ and incubated at 37 °C overnight. Negative control wells did not receive peptide and positive control wells were treated with 1 µg ml^−1^ anti-CD3 (clone OKT3, BioLegend) and 1 µg ml^−1^ anti-CD28 (clone CD28.8, BioLegend) antibodies. ELISPOT assay was performed using manufacturer’s protocol with anti-IFN-γ (clone 1-DK1, Mabtech) capture, biotinylated anti-IFN-γ (clone B6-1, Mabtech) detection, Streptavidin-ALP (Mabtech) and AP Conjugated Substrate (Bio-Rad) followed by disinfection with 0.05% Tween-20 (Thermo Fisher) and analysis using S6 Macro Analyzer (CTL Analyzers). Responses greater than ten spots per well and 3-fold above negative controls were scored as positive^97,96^.

### Assessment of HIV-specific CD8^+^ T cell proliferation

PBMCs were stained at 37 °C for 20 min with 0.5 µM CellTrace carboxyfluorescein succinimidyl ester (CFSE) (Thermo Fisher) as per manufacturer’s protocol at 1 × 10^6^ cells ml^−1^. Staining was quenched with fetal bovine serum (Sigma), and cells were washed twice with R10, resuspended at 1 × 10^6^ ml^−1^ and plated 200 µl per well in 96-well round-bottom polystyrene plates (Corning). Individual HIV peptides corresponding to IFN-γ ELISPOT responses for each participant were added at 1 µg ml^−1^ and incubated at 37 °C for 6 days before flow cytometric assessment. Negative control wells received dimethyl sulfoxide (in amounts equivalent to peptide containing wells), but no peptide. Positive control wells received 1 µg ml^−1^ anti-CD3 (clone OKT3, BioLegend) and anti-CD28 (clone CD28.8, BioLegend) antibodies. On day 6, cells were stained with Live/Dead Violet (Thermo Fisher), PE/Cy7-anti-human CD3 (clone SK7, BioLegend) and APC-anti-human CD8 (clone SK1, BioLegend) and then analyzed by flow cytometry (Supplementary Fig. 4). The frequency of proliferating CD8^+^ T cells was determined by subtracting the percentage of CD8^+^ T cells in the CFSE low gate following HIV peptide stimulation by the highest percentage of CFSE low cells among the dimethyl sulfoxide negative controls.

### HLA typing and HIV escape mutation data analysis

HLA-A/B/C typing was performed using sequence-specific oligonucleotide probing and sequence-based typing as previously described^98^. We excised individual HIV genes from proviral sequences using Gene Cutter^106^. We then identified polymorphisms within these genes that are known to be associated with one or more host HLA alleles expressed, as defined using a published list of HLA-associated polymorphisms across the HIV subtype B proteome^99^. For the escape mutation analysis, each HLA-associated viral site was categorized into one of three groups: (1) ‘non-adapted’ viral sites showed the specific HIV-1 residue predicted to be susceptible to the restricting HLA, (2) ‘adapted’ sites showed the specific HIV-1 residue predicted to confer escape from the restricting HLA, and (3) ‘possibly adapted’ sites showed any residue other than the ‘non-adapted’ form, supporting it as a possible escape variant^100^.

### Limiting dilution VOA

PBMCs from participants and donors without HIV were stimulated with IL-2 (100 U ml^−1^) and PHA (1 µg ml^−1^) for 72 h in R20 culture media. Then, we continued the stimulation with only with IL-2 in R20 culture media. VOAs were performed by using CD4^+^ T cells isolated from cryopreserved PBMCs using EasySep Human CD4^+^ T Cell Enrichment Kit (STEMCELL Technologies) from participants and healthy donors. Then we used MOLT-4/CCR5 cell lines and co-cultured those for more than 30 days, as reported previously^101^. We started with 0.1 × 10^6^ MOLT-4/CCR5 cells and 0.5 × 10^6^ CD4^+^ T cells from our participants and healthy donor and cultured them in each well of a 24-Transwell plates (STEMCELL Technologies). We collected samples from supernatant and MOLT-4/CCR5 every 3 days and refreshed the media with IL-2 (100 U ml^−1^) in R20 ^102,103^.

### Statistical analysis

We analyzed our results by using Mann–Whitney *U* tests (two-tailed), Fisher’s exact tests and Wilcoxon’s tests as appropriate, unless otherwise specified. Correlations were tested by the Spearman’s rank test. Adjustment for multiple comparisons was made in the analysis of ChIP–seq histone marks and host gene transcription, RNA-seq and numbers of HLA escape mutations per HIV-1 gene. A *P* value of less than 0.05 was deemed significant. We adjusted for multiple comparisons in the analysis of ChIP–seq histone marks and host gene transcription, RNA-seq and the number of HLA escape mutations per HIV-1 gene. However, we did not make any corrections for multiple comparisons in the other analyses, as it was an exploratory analysis. We performed the statistical analysis using Prism (GraphPad v.7) and the statistical packages in R (R Project for Statistical Computing, version 4.1.0).

### Reporting summary

Further information on research design is available in the Nature Portfolio Reporting Summary linked to this article.

## Online content

Any methods, additional references, Nature Portfolio reporting summaries, source data, extended data, supplementary information, acknowledgements, peer review information; details of author contributions and competing interests; and statements of data and code availability are available at 10.1038/s41591-023-02611-1.

### Supplementary information

Supplementary Information Supplementary Tables 1–5 and Figs. 1–4.

Reporting Summary

Supplementary Data 1 Primers used in this study.

## Data Availability

All data and code are available by request to the authors and the AIDS Clinical Trials Group. Sequence data were submitted to Genbank (BioProject: PRJNA973660). ROADMAP epigenomic data are available at http://www.roadmapepigenomics.org.

## Code availability

There were no new pipelines or packages generated in this study. CHIP–seq and RNA-seq downstream data analyses code was deposited at https://github.com/yijiali89/NSV.
