# Supplementary material for: Viral and host mediators of non-suppressible HIV-1 viremia
Source: Nat Med. 2023 Nov 13;29(12):3212–23. doi: 10.1038/s41591-023-02611-1 (PMC10719098; doi:10.1038/s41591-023-02611-1)
Supplement: Supplementary file 1 — Supplementary Tables 1–5 and Figs. 1–4. [file 41591_2023_2611_MOESM1_ESM.pdf]

nature medicine

Article

https://doi.org/10.1038/s41591-023-02611-1

Viral and host mediators of non-suppressible HIV-1 viremia

In the format provided by the
authors and unedited

Supplementary information

Supplementary Tables 2

Supplementary Table 1. Characteristics of the non-suppressible viremia (NSV) participants. 2

Supplementary Table 2. Characteristics of the ART-suppressed participants for sequencing analysis. 3

Supplementary Table 3. Characteristics of the ART-suppressed comparator participants for T cell activation and phenotyping. 4

Supplementary Table 4. Characteristics of the viremic controller (VC) comparator participants for T cell activation and phenotyping. 5

Supplementary Table 5. Drug concentrations for NSV participants were quantified using two different methodologies. 6

Supplementary Figures 7

Supplementary Fig. 1. Levels of intracellular emtricitabine-triphosphate (FTC-TP) and tenofovir-diphosphate (TFV-DP) levels in dried blood spots. 7

Supplementary Fig. 2. Neighbor joining tree of all near-full length proviral sequences from NSV participants. 8

Supplementary Fig. 3. Non-suppressible viremia is driven by critical intersection of factors at viral genetic, epigenetic, and cellular level. 9

Supplementary Fig. 4. Gating strategy for CFSE proliferation assay. 10

Supplementary Tables

Supplementary Table 1. Characteristics of the non-suppressible viremia (NSV) participants.

ID	Age Group	Sex	Race	NSV Duration (years)	CD4 cell Count (cells/mm^3)	ART Regimen[a]	GSS[b]
**LV1**	70-79	M	Hispanic	8	390	TAF/FTC/DTG/DRVr	3.25
**LV2**	50-59	M	Native American	2	998	TAF/FTC/DTG/MVC/DRVc	5
**LV3**	60-69	M	White	3	531	TAF/FTC/DTG	2.25
**LV4**	50-59	M	White	1	1472	ABC/3TC/DTG	3
**LV5**	60-69	M	Black	3	598	TAF/FTC/BIC/DOR/DRVr	2.25
**LV7**	60-69	M	White	5	672	TAF/FTC/DTG	3
**LV8**	40-49	F	Black	10	1039	TAF/FTC/BIC	3
**LV9**	50-59	M	White	11	923	TAF/FTC/BIC	3

[a]ART regimen at the time of initial sample collection
[b]GSS, genotypic susceptibility score of plasma virus against the last ART regimen (number of active antiretroviral medications, see Methods section)
ART, antiretroviral therapy; TAF, tenofovir alafenamide; FTC, emtricitabine; DTG, dolutegravir; MVC, maraviroc; DRVr, darunavir-ritonavir; DRVc, darunavir-cobicistat; DOR, doravirine; BIC, bictegravir; ABC, abacavir; 3TC, lamivudine

Supplementary Table 2. Characteristics of the ART-suppressed participants for sequencing analysis.

Age Group	Sex	Race	ART Duration (years)	CD4 cell Count (cells/mm^3)	NFL-seq[a]	RNA-seq[b]
30-39	F	Hispanic	7	1097	Y	N
40-49	M	White	7.7	736	Y	Y
50-59	M	White	7.7	1652	Y	N
50-59	M	Black	6.3	806	Y	N
30-39	M	White	5.4	691	Y	Y
50-59	F	White	5.7	941	Y	N
50-59	F	White	7	781	Y	Y
40-49	F	Black	6.6	685	Y	Y
40-49	M	Black	5.4	1462	Y	N
40-49	M	White	3.2	469	Y	N
30-39	F	White	6.5	1319	N	Y

[a]NFL-seq, near-full length sequencing performed
[b]RNA-seq, bulk CD4+ T cell RNA transcript sequencing performed

Supplementary Table 3. Characteristics of the ART-suppressed comparator participants for T cell activation and phenotyping.

**Age Group**	**Sex**	**Race**	**ART Duration** (years)	**CD4 cell Count** (cells/mm^3)
40-49	M	White	9.25	508
70-79	M	White	9.5	961
60-69	F	Black	14	953
50-59	F	Black	12.5	945
60-69	M	White	13	602
70-79	M	White	13	633
50-59	M	White	11	793

Supplementary Table 4. Characteristics of the viremic controller (VC) comparator participants for T cell activation and phenotyping.

**Age Group**	**Sex**	**Race**	**Viral load** (copies/ml)	**CD4 cell Count** (cells/mm^3)
40-49	M	White	48	496
60-69	M	NA	103	NA
60-69	M	Black	<48	1004
80-89	M	White	160	650
50-59	M	White	396	940
60-69	F	Black	95	838
60-69	F	White	146	1015

Supplementary Table 5. Drug concentrations for NSV participants were quantified using two different methodologies.

a. For two participants LV1 and LV2, antiretroviral concentrations were quantified in plasma b. For other five participants, antiretroviral concentrations were quantified in dried blood spots.

		Plasma Concentration (mcg/mL)							
**PID**	**Current ART**	**TP1[a]**		**TP2**					
		**DTG**	**DRV**	**DTG**	**DRV**				
**LV1**	TAF/FTC/DTG/DRVr	0.29	1.52	2.88	7.21				
**LV2**	TAF/FTC/DTG/DRVr	1.52	6.27	ND[b]	ND				
		**Dried Blood Spots (fmol or pmol/punches)**							
		**TP1**		**TP2**		**TP3**		**LLOQ[c]**	
		**TFV-DP**	**FTC-TP**	**TFV-DP**	**FTC-TP**	**TFV-DP**	**FTC-TP**	**TFV LLOQ**	**FTC LLOQ**
**LV3**	DTG/FTC/TAF	2771	5.1	ND	ND	ND	ND	25	0.1
**LV5**	TAF/BIC/FTC/DOR/DRV	6684	6	ND	ND	ND	ND	25	0.1
**LV7**	FTC/TAF/BIC	3632	6.7	4306	5.2	4941	5.7	25	0.1
**LV8**	FTC/TAF/BIC	3844	4.5	3041	5	3322	4.3	25	0.1
**LV9**	FTC/TAF/BIC	3773	4.5	3480	3.2	ND	ND	25	0.1

[a]TP, time points
[b]ND, Not done
[c]LLOQ, lower limit of detection
"-DP", diphosphate; "-TP", triphosphate.

Supplementary Figures

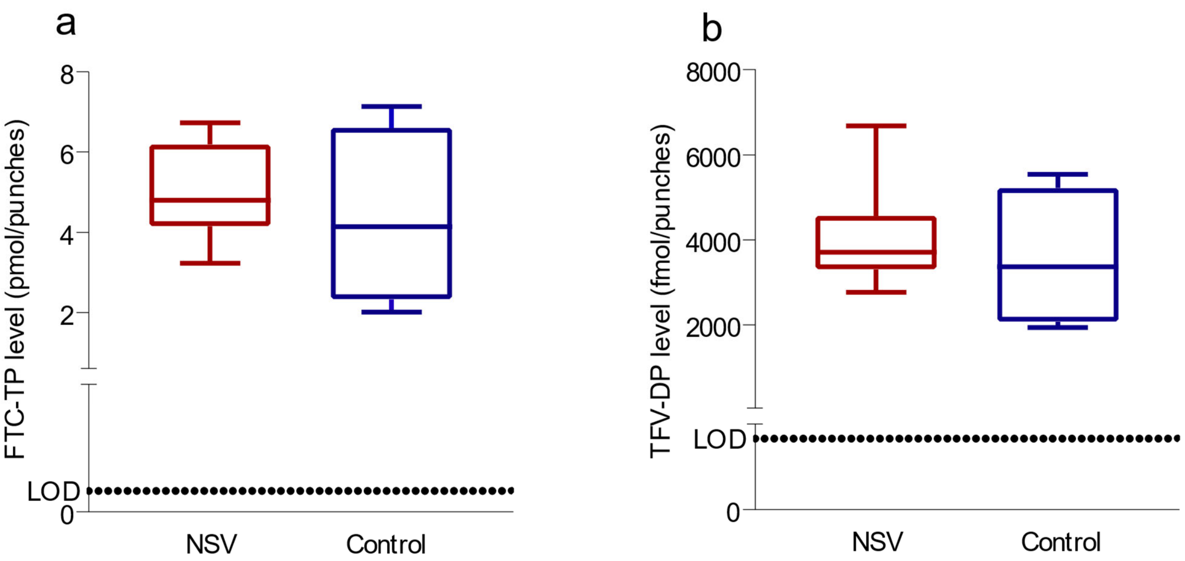

Supplementary Fig. 1. Levels of intracellular emtricitabine-triphosphate (FTC-TP) and tenofovir-diphosphate (TFV-DP) levels in dried blood spots.

(a) Levels of FTC-TP in NSV participants compared to a previously published group of HIV-uninfected individuals receiving daily FTC by directly observed therapy. (b) Levels of TFV-DP in NSV participants compared to a previously published group of ART-suppressed individuals. N=10 biologically independent NSV samples from different time points were compared to n=5 HIV-uninfected samples. Bars show median values and error bars indicate 95% confidence interval. Mann Whitney test was used. Box plot shows median, Q1, Q3, and whiskers show range.

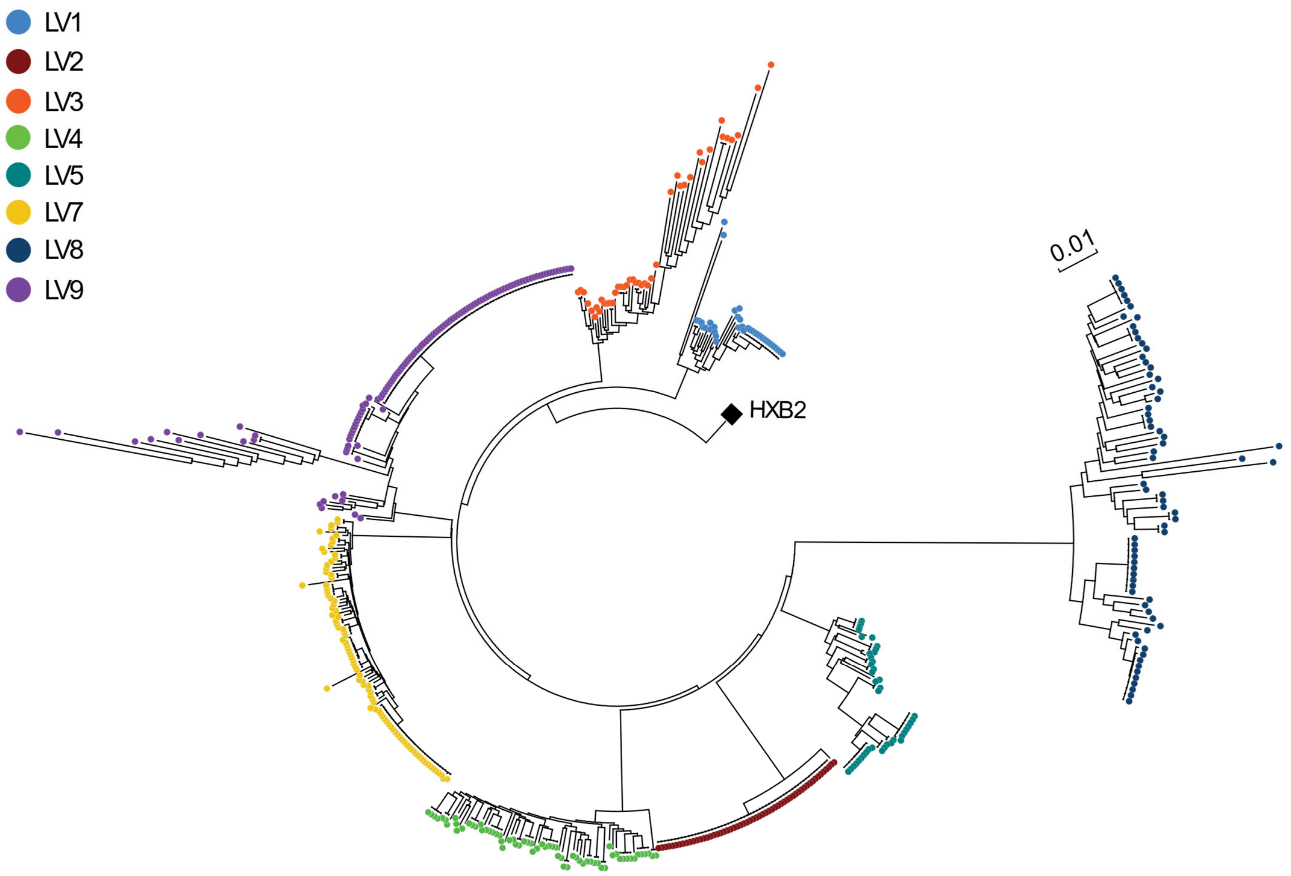

Supplementary Fig. 2. Neighbor joining tree of all near-full length proviral sequences from NSV participants.

The predominant outlier LV8 (dark blue) harbored HIV-1 subtype C virus, while others harbored HIV-1 subtype B virus.

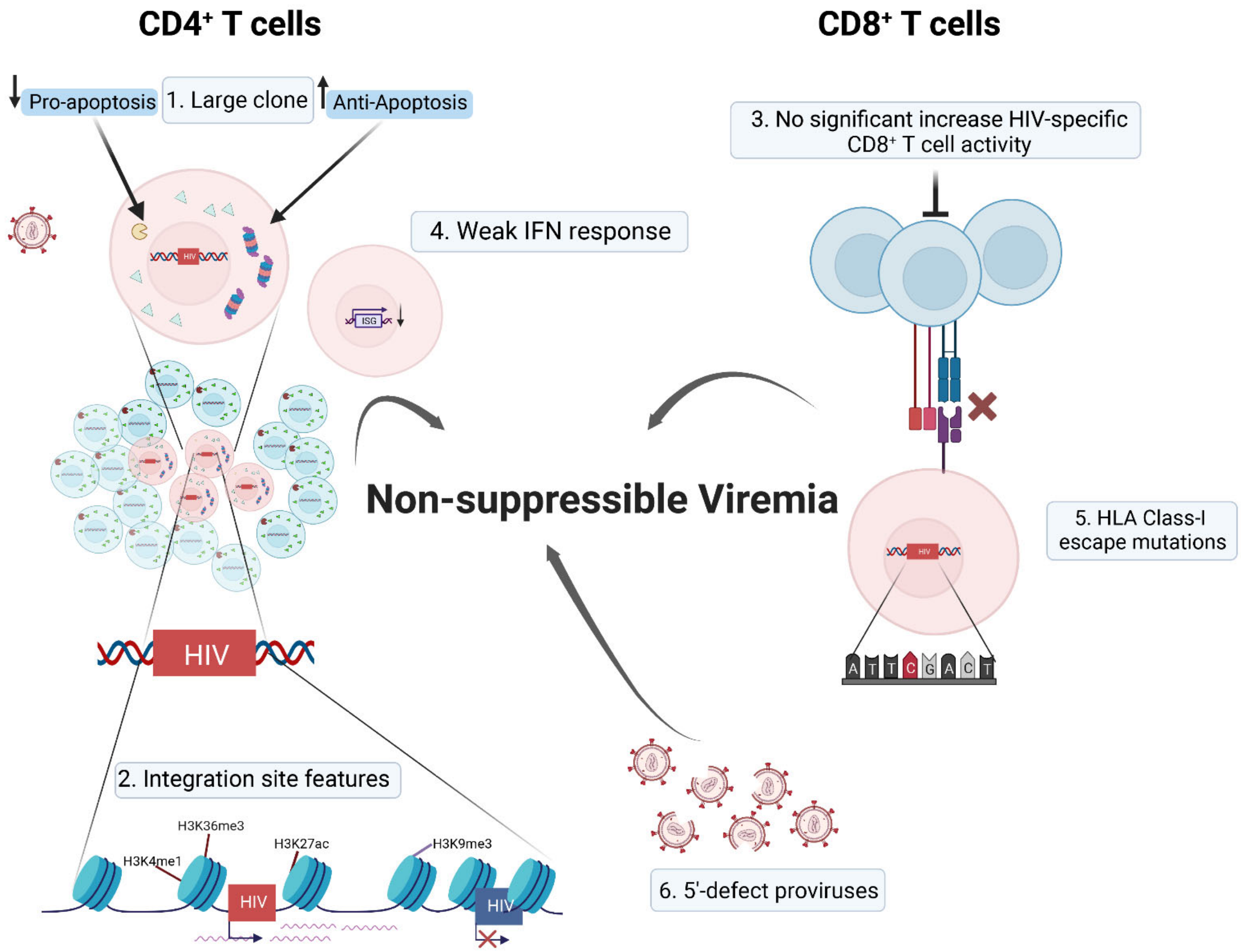

Supplementary Fig. 3. Non-suppressible viremia is driven by critical intersection of factors at viral genetic, epigenetic, and cellular level.

The NSV phenotype was highlighted by the presence of large, clonally-expanded reservoirs of proviruses (1) integrated in transcriptionally-permissive chromosomal regions (2), within a CD4+ T cell environment primed for survival, with a muted HIV-specific immune responses (3, 4), and harboring certain viral mutations (5, 6). Graphics in in this figure were created with Biorender and license was obtained.

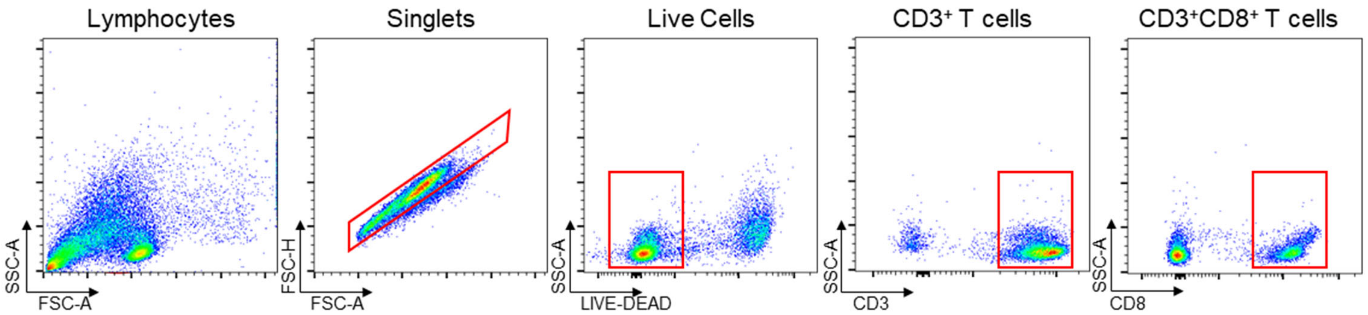

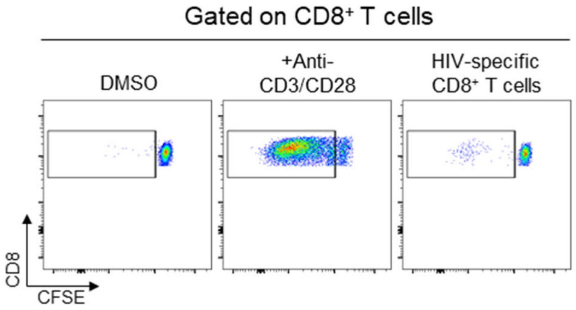

Supplementary Fig. 4. Gating strategy for CFSE proliferation assay.
Representative gating strategy for identification of proliferating CD3+ CD8+ CFSE low T cells in response to HIV peptides. The gate establishing the frequency of CFSE low CD8+ T cells was chosen based on minimizing responses in two negative-control (DMSO) wells and verified using positive control (CD3/CD28) wells.
