# Supplementary material for: Viral and host mediators of non-suppressible HIV-1 viremia
Source: Nat Med. 2023 Nov 13;29(12):3212–23. doi: 10.1038/s41591-023-02611-1 (PMC10719098; doi:10.1038/s41591-023-02611-1)
Supplement: Supplementary file 2 — Reporting Summary [file 41591_2023_2611_MOESM2_ESM.pdf]

Corresponding author(s): Jonathan Li

Last updated by author(s): 09/12/2023

Reporting Summary

Nature Portfolio wishes to improve the reproducibility of the work that we publish. This form provides structure for consistency and transparency in reporting. For further information on Nature Portfolio policies, see our Editorial Policies and the Editorial Policy Checklist.

Statistics

For all statistical analyses, confirm that the following items are present in the figure legend, table legend, main text, or Methods section.

n/a	Confirmed	
☐	☒	The exact sample size (*n*) for each experimental group/condition, given as a discrete number and unit of measurement
☐	☒	A statement on whether measurements were taken from distinct samples or whether the same sample was measured repeatedly
☐	☒	The statistical test(s) used AND whether they are one- or two-sided<br>*Only common tests should be described solely by name; describe more complex techniques in the Methods section.*
☒	☐	A description of all covariates tested
☐	☒	A description of any assumptions or corrections, such as tests of normality and adjustment for multiple comparisons
☐	☒	A full description of the statistical parameters including central tendency (e.g. means) or other basic estimates (e.g. regression coefficient) AND variation (e.g. standard deviation) or associated estimates of uncertainty (e.g. confidence intervals)
☐	☒	For null hypothesis testing, the test statistic (e.g. *F*, *t*, *r*) with confidence intervals, effect sizes, degrees of freedom and *P* value noted<br>*Give P values as exact values whenever suitable.*
☒	☐	For Bayesian analysis, information on the choice of priors and Markov chain Monte Carlo settings
☒	☐	For hierarchical and complex designs, identification of the appropriate level for tests and full reporting of outcomes
☒	☐	Estimates of effect sizes (e.g. Cohen's *d*, Pearson's *r*), indicating how they were calculated

Our web collection on statistics for biologists contains articles on many of the points above.

Software and code

Policy information about availability of computer code

Data collection	ROADMAP epigenomic data (available at http://www.roadmapepigenomics.org)
Data analysis	Prism (GraphPad version 8.2.1) (https://www.graphpad.com/scientific-software/prism/, version 8.2.1)<br>R (version 4.1.0) (https://www.r-project.org, version 4.1)<br>Tidyverse (https://tidyverse.tidyverse.org, version 2.0.0)<br>ggpubr (https://rpkgs.datanovia.com/ggpubr/, version 0.6.0)<br>ggsci (https://github.com/nanxstats/ggsci, version 3.0.0)<br>dunn.test (https://CRAN.R-project.org/package=dunn.test, version 1.3.5)<br>Gene cutter: https://www.hiv.lanl.gov/content/sequence/GENE_CUTTER/cutter.html (no version available, last modified: Thu Sep 26 13:20 2019)<br>MAFFT v7.2.0 (https://mafft.cbrc.jp/alignment/server/)<br>MEGA 6 (https://www.megasoftware.net/)<br>Proviral intactness pipeline (no version number available, https://github.com/BWH-Lichterfeld-Lab/Intactness-Pipeline)<br>The Stanford HIV database (Version 9.4)<br>VIPER pipeline (no version number, https://bitbucket.org/cfce/viper/src/master/)<br>DESeq2 package (version 1.40.2)<br>Integration site analysis (no version number): https://indra.mullins.microbiol.washington.edu/integrationsites/<br>nearestTSS (from edgeR v3.42): Find Nearest Transcriptional Start Site<br>S6 Macro Analyzer (Immunospot 7.0.38.3 Professional DC, Cellular Technology Ltd.)<br>Adobe Illustrator (https://www.adobe.com/products/illustrator.html, version 2022 (26.1))<br>Biorender (https://biorender.com)

PMCMRplus (version 1.9.7)
Cytoscape version 3.10.1
FlowJo Version 9.0.1 (http://flowjo.com)
fgsea (version 1.26.0)
BD FACSDiva (version 8.0.2)

For manuscripts utilizing custom algorithms or software that are central to the research but not yet described in published literature, software must be made available to editors and reviewers. We strongly encourage code deposition in a community repository (e.g. GitHub). See the Nature Portfolio guidelines for submitting code & software for further information.

Data

Policy information about availability of data

All manuscripts must include a data availability statement. This statement should provide the following information, where applicable:

- Accession codes, unique identifiers, or web links for publicly available datasets
- A description of any restrictions on data availability
- For clinical datasets or third party data, please ensure that the statement adheres to our policy

All data are available by request. Correspondence and requests for materials should be addressed to Dr. Jonathan Li (jli@bwh.harvard.edu).

Human research participants

Policy information about studies involving human research participants and Sex and Gender in Research.

Reporting on sex and gender	We have reported the sex and race in Table 1 of the manuscript
Population characteristics	Population characteristics are reported in Table 1 of the manuscript.
Recruitment	We enrolled 8 ART-treated participants with ≥3 HIV-1 RNA levels between 40-1000 copies/mL over 24 months and compared them to a group of ART-suppressed participants and viremic controllers with similar demographic and HIV characteristics. The ART-suppressed and viremic controller comparators included participants from the AIDS Clinical Trials Group (ACTG) and the Ragon Institute of Mass General, MIT and Harvard. Sex and/or gender of participants was determined based on self-report.
Ethics oversight	All study participants provided written informed consent. The study was approved by the Mass General Brigham Institutional Review Board.

Note that full information on the approval of the study protocol must also be provided in the manuscript.

Field-specific reporting

Please select the one below that is the best fit for your research. If you are not sure, read the appropriate sections before making your selection.

☒ Life sciences ☐ Behavioural & social sciences ☐ Ecological, evolutionary & environmental sciences

For a reference copy of the document with all sections, see nature.com/documents/nr-reporting-summary-flat.pdf

Life sciences study design

All studies must disclose on these points even when the disclosure is negative.

Sample size	A total of n=8 ART-treated participants with ≥3 HIV-1 RNA levels between 40-1000 copies/mL over 24 months called non-suppressible HIV-1 viremia (NSV), n=11 ART-suppressed participants from the AIDS Clinical Trials Group (ACTG), n=7 ART-suppressed participants from the Ragon Institute of MGH, MIT and Harvard and n=7 viremic controllers from the Ragon Institute of Mass General, MIT and Harvard. Written informed consent was obtained from all participants. Participants were analyzed in data described in Figure 1-5.<br><br>Sample size was not calculated and was determined based on sample availability, as NSV is not an extremely common event and there is certain difficulty to recruit and collect sample from PWH with NSV. Previous studies on this topic usually included less than 5 sample and we included 8 here. We are not aware of significant self-selection bias.
Data exclusions	No data were excluded
Replication	Large clonal clusters were confirmed with multiple identical proviral and plasma RNA sequences. Positive and negative controls were included in the experiments.
Randomization	No randomization was performed as participants were already assigned as NSV or virally suppressed based on their clinical viral load data.

Blinding | No blinding was performed and sample names were labeled based on their de-identified ID, sample collection date and time points. No blinding was done to avoid sample cross-contamination.

Reporting for specific materials, systems and methods

We require information from authors about some types of materials, experimental systems and methods used in many studies. Here, indicate whether each material, system or method listed is relevant to your study. If you are not sure if a list item applies to your research, read the appropriate section before selecting a response.

Materials & experimental systems

n/a	Involved in the study
☐	☒ Antibodies
☐	☒ Eukaryotic cell lines
☒	☐ Palaeontology and archaeology
☒	☐ Animals and other organisms
☐	☒ Clinical data
☒	☐ Dual use research of concern

Methods

n/a	Involved in the study
☒	☐ ChIP-seq
☐	☒ Flow cytometry
☒	☐ MRI-based neuroimaging

Antibodies

Antibodies used

Live/Dead Violet (Thermo Fisher Cat# L34954, Lot# 2615872, 1:1000)
PE-Cy7-CD3 (clone SK7, Biolegend Cat# 344816, Lot# B385424, 1:100),
BV711-anti-CD4 (clone RPA-T4, Biolegend Cat# 300558, Lot# B354515, 1:100)
APC-anti-CD8 (clone SK1, Biolegend Cat# 344722, Lot# B340556, 1:100)
Alexa Fluor 700-anti-CD25 (clone M-A251, Biolegend Cat# 356118, Lot# B302029, 1:50)
BV650-anti-CD38 (clone HB-7, Biolegend Cat# 356619, Lot# B370845, 1:50)
FITC-anti-CD69 (FN50, Biolegend Cat# 310904, Lot# B290844, 1:50)
BV785-anti-HLA-DR (clone L243, Biolegend Cat# 307641, Lot# B367913, 1:50)
PE/Dazzle 594-anti-PD-1 (clone EH12.2H7, Biolegend Cat# 329940, Lot# B367981, 1:100)
Carboxyfluorescein succinimidyl ester (CFSE; Life Technologies Cat# C34554, Lot# 2633292, 0.5 μM)

Validation

Antibodies are routinely used for highly expressed cell surface markers

Eukaryotic cell lines

Policy information about cell lines and Sex and Gender in Research

Cell line source(s)

MOLT-4/CCR5 cell line were used for viral outgrowth. Cell lines were obtained directly from the NIH AIDS Reagent Program (https://www.niaid.nih.gov/research/nih-aids-reagent-program and https://www.hivreagentprogram.org/Catalog/HRPCellLines/ARP-4984.aspx)

Authentication

Cell lines were obtained directly from the NIH AIDS Reagent Program, which authenticates and distributes HIV reagents (https://www.niaid.nih.gov/research/nih-aids-reagent-program). We further authenticate the cell line based on morphology.

Mycoplasma contamination

No contamination by mycoplasma was observed

Commonly misidentified lines
(See ICLAC register)

No commonly misidentified cell lines were used

Clinical data

Policy information about clinical studies

All manuscripts should comply with the ICMJE guidelines for publication of clinical research and a completed CONSORT checklist must be included with all submissions.

Clinical trial registration

NA

Study protocol

Note where the full trial protocol can be accessed OR if not available, explain why.

Data collection

NA

Outcomes

NA

Flow Cytometry

Plots

Confirm that:

☒ The axis labels state the marker and fluorochrome used (e.g. CD4-FITC).

☒ The axis scales are clearly visible. Include numbers along axes only for bottom left plot of group (a 'group' is an analysis of identical markers).

☒ All plots are contour plots with outliers or pseudocolor plots.

☒ A numerical value for number of cells or percentage (with statistics) is provided.

Methodology

Sample preparation	PBMCs were stained at 37°C for 20 minutes with 0.5 µM CellTrace CFSE (Thermo Fisher) as per manufacturer's protocol at 1x106 cells/mL. Staining was quenched with FBS (Sigma), cells were washed twice with R10, resuspended at 1x106/mL and plated 200 µL per well in 96-well round-bottom polystyrene plates (Corning). Individual HIV peptides corresponding to IFN-γ ELISPOT responses for each patient were added at 1 µM and incubated at 37°C for 6 days before flow cytometric assessment. Negative control wells did not receive peptide and positive control wells received 1 µg/mL anti-CD3 (clone OKT3, Biolegend) and anti-CD28 (clone CD28.8, Biolegend) antibodies. On day 6, cells were stained for viability using Live/Dead Violet (Thermo Fisher), AlexaFluor700-anti-CD3 (clone SK7, Biolegend), BUV395-anti-CD8 (clone RPA-T8, BD Biosciences), and APC-pHLA tetramer matching the peptide used for stimulation, then analyzed by flow cytometry.<br><br>For activation marker staining, peripheral blood mononuclear cells (PBMC) were thawed and stained for 20 min at room temperature with Live/Dead Violet (Thermo Fisher), PE-Cy7-CD3 (clone SK7, Biolegend), BV711-anti-CD4 (clone RPA-T4, Biolegend), APC-anti-CD8 (clone SK1, Biolegend), Alexa Fluor 700-anti-CD25 (clone M-A251, Biolegend), BV650-anti-CD38 (clone HB-7, Biolegend), FITC-anti-CD69 (FN50, Biolegend), BV785-anti-HLA-DR (clone L243, Biolegend), PE/Dazzle 594-anti-PD-1 (clone EH12.2H7, Biolegend). Cells were washed and fixed in 2% paraformaldehyde prior to flow cytometric analysis on a BD LSR Fortessa (BD Biosciences). CD4 surface expression was determined by assessment of CD4 mean fluorescence intensity (MFI).
Instrument	BD Biosciences LSR II
Software	BD Facs Diva was used for data acquisition. FlowJo Version 9.0.1 was used to analyze the data.
Cell population abundance	The relevant CD3+CD8+CFSElow T cells ranged from 0.1-7% of CD3+CD8+ T cells
Gating strategy	For CFSE-based proliferation assays, we used the following gating strategy: FSC-A/SSC-A (lymphocytes), FSC-A/FSC-H (single cells),Viability Dye/SSC-A (live cells), CD3/SSC-A (CD3+ T cells), CD8/SSC-A (CD3+ CD8+ T cells), CD8/CFSE (CD3+CD8+CFSElow T cells).<br><br>For activation marker staining, FSC-A/SSC-A (lymphocytes), FSC-A/FSC-H (single cells),Viability Dye/SSC-A (live cells), CD3/SSC-A (CD3+ T cells), CD4/CD8 (CD3+CD4+ or CD3+CD8+ T cells), CD38/HLA-DR (CD3+CD4+CD38+HLA-DR+ or CD3+CD8+CD38 +HLA-DR+ T cells, activated T cells)

☒ Tick this box to confirm that a figure exemplifying the gating strategy is provided in the Supplementary Information.
